# Integrated Omics and Computational Glycobiology Reveal Structural Basis for Influenza A Virus Glycan Microheterogeneity and Host Interactions*[Fn FN2]

**DOI:** 10.1074/mcp.M116.058016

**Published:** 2016-03-16

**Authors:** Kshitij Khatri, Joshua A. Klein, Mitchell R. White, Oliver C. Grant, Nancy Leymarie, Robert J. Woods, Kevan L. Hartshorn, Joseph Zaia

**Affiliations:** From the ‡Center for Biomedical Mass Spectrometry, Department of Biochemistry, Boston University School of Medicine, Boston, Massachusetts 02118;; §Bioinformatics Program, Boston University, Boston, Massachusetts 02215;; ¶Department of Medicine, Boston University School of Medicine, Boston, Massachusetts 02118;; ‖Complex Carbohydrate Research Center, University of Georgia, Athens, Georgia 30602

## Abstract

Despite sustained biomedical research effort, influenza A virus remains an imminent threat to the world population and a major healthcare burden. The challenge in developing vaccines against influenza is the ability of the virus to mutate rapidly in response to selective immune pressure. Hemagglutinin is the predominant surface glycoprotein and the primary determinant of antigenicity, virulence and zoonotic potential. Mutations leading to changes in the number of HA glycosylation sites are often reported. Such genetic sequencing studies predict at best the disruption or creation of sequons for *N*-linked glycosylation; they do not reflect actual phenotypic changes in HA structure. Therefore, combined analysis of glycan micro and macro-heterogeneity and bioassays will better define the relationships among glycosylation, viral bioactivity and evolution. We present a study that integrates proteomics, glycomics and glycoproteomics of HA before and after adaptation to innate immune system pressure. We combined this information with glycan array and immune lectin binding data to correlate the phenotypic changes with biological activity. Underprocessed glycoforms predominated at the glycosylation sites found to be involved in viral evolution in response to selection pressures and interactions with innate immune-lectins. To understand the structural basis for site-specific glycan microheterogeneity at these sites, we performed structural modeling and molecular dynamics simulations. We observed that the presence of immature, high-mannose type glycans at a particular site correlated with reduced accessibility to glycan remodeling enzymes. Further, the high mannose glycans at sites implicated in immune lectin recognition were predicted to be capable of forming trimeric interactions with the immune-lectin surfactant protein-D.

Influenza A virus (IAV)^1^ remains a major cause of animal and human mortality and morbidity, with no effective broadly neutralizing vaccines available. The IAV envelope-glycoprotein hemagglutinin (HA) binds sialic acids on respiratory airway surface glycoconjugates and facilitates viral entry into host cells, thereby leading to infection. The envelope-glycoprotein neuraminidase (NA) cleaves sialic acids to allow newly formed virions to escape the host cell membrane. Because the surface proteins undergo rapid mutation, it has not been possible to develop broadly neutralizing vaccines against IAV. In particular, IAV strains undergo amino acid mutations to accumulate new *N*-glycosylation sequons during seasonal circulation in the human population (1–4). Thus, based primarily on genetic sequencing evidence (5–7), glycans appear to shield HA antigenic sites from host antibody recognition, as part of the evolutionary antigenic drift (8–11). Although this putative shielding effect of glycans apparently improves the ability of the virus to escape antibody neutralization, the virus must compensate for loss in receptor avidity from steric interference caused by the glycans proximal to the sialic acid binding sites on the HA head (12, 13).

T-cells of the adaptive immune system reach the lung approximately 5 days after IAV infection (14). During this time, viral replication and lung damage is mitigated by the innate immune system. Among the soluble innate immune inhibitors, the calcium-dependent lectin inhibitors (known as β-inhibitors) bind to carbohydrates on pathogen surfaces. For seasonal IAV strains, surfactant protein-D (SP-D) is the most important soluble innate immune factor present in bronchial lavage fluid (15, 16). The activity of this lectin depends on the presence of high mannose *N*-glycans on viral HA; IAV strains lacking glycosylation on the HA head region resist neutralization by SP-D. These include the pandemic strains of H1N1 1918 and 2009, the H3N2 of 1968 and the H2N2 of 1957 (17–19). Thus, the pathogenicity of pandemic IAV correlates with ability to escape neutralization by SP-D (15, 18, 20). Generally speaking, as seasonal strains acquire additional *N*-glycosylation sequons they show increased susceptibility to SP-D, as they circulate in humans. Although IAV mutates to avoid antibody neutralization by accumulating sequons, the mechanisms whereby viruses maintain fitness despite increased binding to SP-D and other lectins of the innate immune system remain poorly understood (16, 19, 21–25).

The H3N2 subtype has circulated in humans since 1968 and remains the major seasonal IAV health threat. The H1N1 subtype was introduced into the human population with the disastrous 1918 pandemic and was supplanted by the H2N2 subtype in 1957. The H1N1 subtype was re-introduced into the human population in 1977 and displayed several additional *N*-glycosylation sequons on HA not present on earlier strains (26). In 2009, it underwent antigenic shift by incorporation of genetic elements from the swine and avian sources to emerge as a new pandemic subtype. The pattern of *N*-glycosylation sequons of pandemic H1N1 2009 more closely resembled the 1918 pandemic H1N1 (minimal glycosylation) than that circulating seasonally prior to 2009 (4).

Despite the central role of glycosylation in viral evolution, there remains a paucity of structural information on HA glycosylation. A recent study of engineered variants of the Hong Kong 1968 H3N2 subtype demonstrated that exclusively high-mannose *N*-glycans occupy HA sites 165 and 246 (27). Although genetic sequence analyses identify the creation or disruption of *N*-glycosylation sequons, they provide no information on the glycan site occupancy, structure and micro-heterogeneity. In addition, crystal structures typically show only the chitobiose core and do not define glycan antennae (28–30). We therefore built a predictive model for seasonal IAV fitness and antigenicity that incorporates (1) data on changes in site-specific HA glycosylation that occur as the virus evolves to escape SP-D neutralization and (2) the HA structural features that regulate glycan microheterogeneity at each site that influences binding to SP-D. We demonstrate the value of complete information on the mature glycosylated HA structures to inform better understanding of the evolution of IAV in response to immune system pressure.

## EXPERIMENTAL PROCEDURES

### 

#### 

##### Virus Preparation

The following IAV strains were used in the study:

##### Phil-82

A high growth reassortant of A/Philippines/2/82(H3N2) and A/Puerto Rico/8/34(H1N1). The seed stock was a gift from Dr. E. Margot Anders, University of Melbourne. This strain consisted of an H3 hemagglutinin and N2 neuraminidase from A/Philippines/2/82, whereas the remaining virus components were from A/Puerto Rico/8/34.

##### Phil-BS

A mutant version of Phil-82 that was selected by growing Phil-82 in the presence of bovine serum, found to be resistant to neutralization by bovine conglutinin, as described previously (22–24, 31). The seed stock was from Dr. E. Margot Anders, University of Melbourne.

##### PR-08

The mouse-adapted A/Puerto Rico/8/34(H1N1) strain. The seed stock was from Dr. Jon S. Abramson, Wake Forest University.

All viruses were grown in the chorioallantoic cavity of 10 day old embryonated chicken eggs. Allantoic fluid was harvested after incubating for 48 h and insoluble debris were pelleted by centrifugation at 1000 × *g*. The virions were then pelleted at 135,000 × *g* and the pellets were purified on a sucrose density gradient followed by dialysis against phosphate buffered saline (PBS).

##### Experimental Design and Statistical Rationale

**Fig. 1.**
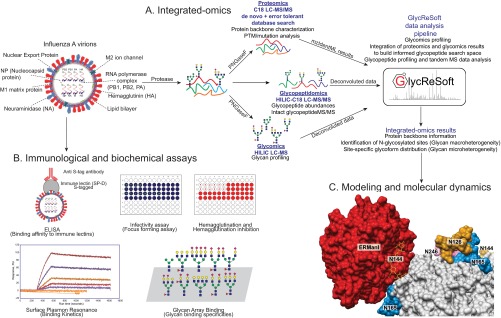
**Experimental Workflow.** *A*, Integrated-omics: Workflow for acquiring and combining proteomics, glycomics and glycopeptidomics information to enable confident assignment of site-specific glycoforms; *B*, Immunological and biochemical assays used for correlating changes in virus glycosylation with bioactivity; *C*, Modeling and molecular dynamics simulations driven by structural information from integrated-omics analyses to understand structural basis for host-virus interactions and glycan processing at important sites. Graphic shows ERManI interacting with the glycan at Asn^144^ on the head region of a Phil-82 hemagglutinin trimer.

As shown in Fig. 1, virus samples were subjected to proteolytic digestion. Proteolytic samples were divided into three fractions for proteomics, glycomics and glycoproteomics. Proteomics and glycomics fractions were subjected to PNGase *F* glycan release. All three fractions for each sample were subjected to triplicate LC-MS analyses, as described in sections below. Three technical replicates of LC-MS acquisitions were used to calculate mean analyte abundances and standard deviations in measurements. Human α-1-acid glycoprotein (Sigma-Aldrich, St. Louis, MO), a widely studied standard glycoprotein, was used as a control for all sample preparation and verifying LC-MS instrument performance.

##### LC-MS Analyses

##### Sample Preparation

Virus preparations were subjected to membrane disruption, reduction, alkylation and proteolysis. Virus preparations containing ∼60 μg total protein were dried down in a centrifugal evaporator and re-suspended in LC-MS grade methanol (Fisher Scientific), followed by sonication for 10 min in a water bath to disrupt viral membranes. Samples were dried and re-suspended in 50% 100 mm ammonium bicarbonate and 50% 2, 2, 2-trifluoro ethanol (TFE) (Sigma-Aldrich) (60 μl). 200 mm dithiothreitol (DTT) (2 μl) was added to the samples and heated at 95 °C for 30 mins to reduce disulfide bonds. Iodoacetamide (10 μl, 200 mm in water) was then added to the samples and incubated for 1 h in dark, at room temperature. To quench excess iodoacetamide, another (2 μl) of 200 mm DTT was added to the samples and incubated for one hour at room temperature. LC-MS water (300 μl) was added to the samples to dilute TFE to less than 5% of the total volume and 100 mm ammonium bicarbonate (200 μl) was added to raise pH to 7.5. Sequencing grade trypsin or chymotrypsin (Promega Corp., Madison, WI) were added to the samples and incubated overnight at 37 °C, for proteolysis. Proteolytic peptides and glycopeptides were split into aliquots for performing ^16^O-proteomics, ^18^O-proteomics (for site-occupancy analysis) and glycoproteomics. ^16^O-proteomics and ^18^O-proteomics samples were deglycosylated using PNGase *F* (New England Biolabs, Ipswitch, MA) in H_2_^16^O or H_2_^18^O respectively, whereas the glycoproteomics samples were incubated in the deglycosylation conditions without PNGase*F*. Released glycans from deglycosylated samples were isolated using C18 spin columns (Pierce Biotechnology, Rockford, IL) and pooled (^16^O and ^18^O) for glycomics analyses, and deglycosylated peptides were eluted and collected separately for proteomics analyses. As a control for spontaneous deamidation at inherently nonglycosylated asparagine residues, the glycosylated samples (no PNGase *F* treatment) were also analyzed, using the proteomics workflow. Mass spectrometry data were acquired on each sample as three technical replicates, unless otherwise stated.

##### Proteomics Analyses

Deglycosylated peptides were subjected to LC-MS/MS using a Waters™ NanoAcquity™ nano-flow chromatograph (Waters Corp., Milford, MA) mounted with a Waters™ Xbridge™ reversed-phase column (150 μm × 100 mm) packed with 1.7 μm BEH C18 resin and a Waters™ trap column (180 μm × 20 mm) packed with 5 μm Symmetry™ C18 stationary phase. The chromatography was performed online with a Q-Exactive Plus™ mass spectrometer (Thermo Fisher Scientific™, San Jose, CA), operated in positive ion mode. The mass spectrometer was mounted with an Advion NanoMate (Advion Inc., Ithaca, NY) source for introduction of the LC eluate by nano-ESI. The source was operated at 1.7 kV with transfer-capillary temperature maintained at 250 °C and S-Lens RF level set at 55. Mass spectra were acquired in the Orbitrap mass analyzer with 1 microscan per spectrum for both MS and MS/MS. Resolving power for MS and MS/MS were set at 70,000 and 17,500, respectively. Tandem MS data were acquired in parallel with MS, on the top 20 most abundant multiply charged precursors, with higher energy collisional dissociation (HCD) at normalized collision energy of 27V. Precursors were isolated using a 1.4 Th window and dynamic exclusion of 10 s was enabled during precursor selection.

##### Glycomics Analyses

Released *N*-glycans were desalted using a Superdex™ Peptide™ 3.2/30 size exclusion column on a Beckman™ Gold chromatograph. Desalted *N*-glycans were analyzed in triplicate using negative mode hydrophilic interaction liquid chromatography (HILIC) -MS with a constant acetonitrile post-column make-up-flow on an Agilent™ 6520 LC-MS system with chip-cube™ nanoESI source, as described previously (32).

##### Glycoproteomics Analyses

Entire tryptic digests were subjected to triplicate analyses using chip-LC-MS/MS with online HILIC enrichment and C18 separation on an Agilent™ 6550 LC-MS system with chip-cube™ ionization source as described previously (33) and by C18 LC-MS/MS without any enrichment on the Q-Exactive Plus™ instrument as described for the proteomics samples. Chymotryptic samples were analyzed only using C18 LC-MS/MS on the Q-Exactive Plus™.

##### Data Analysis

##### Proteomics

Proteomics data were subjected to PEAKS SPIDER (*de novo* + database + error tolerant PTM) searches in PEAKS (34) Studio 7.5 (Bioinformatics Solutions Inc., Waterloo, ON) against a combined Uniprot (35) and Influenza A virus protein database (36). The FASTA sequence database used for searches has been included in the data repository. A 50 ppm error tolerance for the precursor (MS^1^) and 0.1 Da mass error tolerance for fragment ions (MS^2^) were specified. A maximum of 2 missed cleavages per peptide were allowed for the database search. Trypsin specificity was defined as cleavage after Arg and Lys, when not followed by a Pro. Chymotrypsin cleavage specificity was after Phe, Leu, Met, Trp and Tyr residues, when not followed by a Pro. Cysteine carbamidomethylation was specified as a fixed modification; deamidation (Asn) and oxidation (Met) were specified as variable modifications. ^18^O labeling of deglycosylated asparagine was also used as a variable modification in case of ^18^O-proteomics samples. After a regular database search, an error-tolerant PTM search was also performed searching for a larger subset of modifications from Unimod (37) and any amino acid substitutions. The final results were a combination of database, *de novo* and error-tolerant searches. False discovery rates (FDR) were calculated using a decoy-fusion approach in PEAKS 7.5, as described previously (34). Identified peptide-spectrum-matches with −10logP value of 15 or higher were kept, at a FDR threshold of 0.1%.

##### Site-occupancy Analysis

Site-occupancy analysis was performed on Phil-82 for glycan modeling and molecular dynamics. Although ^18^O-proteomics was performed to define glycan site-occupancy, in cases where multiple asparagine and glutamine residues existed on peptides containing the *N*-glycosylation sequons, spontaneous deamidation of these residues in presence of H_2_^18^O, confounded the data. Therefore, ^16^O-proteomics results were instead used to define site-occupancy information.

From the proteomics search results, peptides spanning glycosylation sites were selected and extracted ion chromatograms were integrated to generate chromatographic peak areas for paired analyses of unoccupied peptides bearing glycosylation sequon in glycosylated samples and the same peptides in PNGase*F* deglycosylated sample. Average peak areas of three replicate analyses were compared after normalization using a high-confidence peptide from a different protein identified across runs. Site-occupancy for sites 8 and 22 were calculated from proteomics results of chymotryptic digests, whereas for the remaining sites, proteomics results from tryptic samples were used.

Percent site occupancy was calculated as

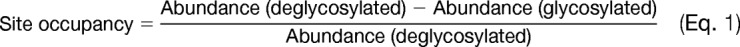


##### Glycomics

Negative mode HILIC-MS data were deconvoluted and deisotoped using the THRASH algorithm in DeconTools/Decon2LS (version 1.0.5501) (38, 39). The deconvoluted/deisotoped peaklists were matched against a theoretical composition hypothesis containing human and avian *N*-linked glycans from GlycomeDB (40, 41), using GlycReSoft (42). Up to 2 formate adducts were allowed while searching for glycan compositions, with a match error tolerance of 20 ppm in the theoretical masses. Abundances of different adducted forms were grouped together and reported as mean of three technical replicates.

##### Glycoproteomics/Integrated-omics

Glycopeptide LC-MS/MS datawere deconvoluted and deisotoped using DeconTools (39) for MS^1^ spectra. Data were converted to mzML format (43) using the MSConvert tool in Proteowizard (version 3.0.7692) (44) and the MS^2^ spectra were then deconvoluted using MasSpike (45). Deconvoluted data were analyzed using an in-house version of GlycReSoft and validated manually. Source code for GlycReSoft is publically hosted on GitHub (https://github.com/GlycReSoft2). Naïve and informed glycopeptide hypotheses were created for mining glycopeptide data. For naïve hypothesis generation, theoretical digests of glycoproteins were generated for combining *N*-glycosylation sequon containing peptides with *N*-glycan compositions from GlycomeDB (41) glycan databases.

For informed hypothesis generation, proteomics search results exported in mzIdentML 1.1 format, were used. Hemagglutinin peptides that contained Asn from the *N*-linked glycosylation sequon, with −10logp values of 20 or higher were included in the hypotheses. Peptide variants were combined with glycoforms identified in glycomics data, to build the glycoproteomics hypotheses. MS^1^ deconvoluted data were searched against glycopeptide hypotheses, using a match error-tolerance of 20 ppm. Theoretical fragment ions were generated for the MS^1^ matches and then searched against the MS^2^ deconvoluted peak lists, using a 20 ppm error tolerance. Features searched for in MS^2^ data of glycopeptides included oxonium ions, peptide backbone ions, peptide backbone ions with an attached HexNAc and stub-glycopeptide ions (33). A glycopeptide spectrum match, was required to have mono or disaccharide oxonium ions. Automated glycopeptide analysis results have been included in the data repository. MS^1^ scores were calculated as described previously (42). MS^2^ scores are representative of number of fragment ions found (peptide backbone and stub ion coverage) (33). q-values depict minimal FDR threshold at which the identification is accepted, as described by Käll *et al.* (46). For cases, where more than one glycopeptide composition matched a given precursor and fragment ion spectrum, only the highest scoring match was retained for reporting in the results. Glycopeptide analysis results represent site-specific glycoform abundances for glycopeptides assigned confidently based on MS^2^ spectra by GlycReSoft and validated manually. Chymotryptic glycopeptide data were manually analyzed for resolving glycoforms on Asn^8^ and Asn^22^ on Phil-82 and Phil-BS and integrated with site-specific glycosylation data from tryptic samples processed using GlycReSoft.

##### Bioassays

Biochemical and immunological assays were performed to correlate changes in site-specific glycan distributions in IAV strains with virus fitness and susceptibility toward host innate immune system. SP-D is a collectin produced and secreted by lung epithelial cells in humans and other mammals. SP-D is also known to inhibit IAV by binding its surface glycoproteins HA and NA (15, 31, 20, 47). For this study, a recombinant full length human SP-D (rhSPDII) that exists as dodecamers was prepared, as described previously by Nikolaidis *et al.* (48, 49). In addition to the full-length SP-D, recombinant neck and carbohydrate recognition domain (NCRD) from SP-D were also used. These NCRDs lack the *N*-terminal domain and the extended collagen domain present in the full-length SP-D and as a result form homotrimers instead of higher order multimers. The wild-type recombinant human NCRD (huNCRD) has been shown to bind glycans but lacks antiviral activity because of absence of cooperative binding effect from the multiple heads (49–51). Gain-of-function mutants R343V and double mutant D325A+R343V (D+R), which have been shown to possess increased virus binding and neutralizing activity (48, 49, 52–54), were also used in bioassays. Mutant names are indicative of amino acid substitutions flanking the lectin site in the huNCRD protein.

##### Hemagglutination Assays

Hemagglutination and hemagglutination inhibition (HI) assays were performed in round bottom 96 well plates, using PBS containing calcium and magnesium (PBS++) as a diluent. Viral titers were first measured using hemagglutination assays, performed as described previously (55), using type O- red blood cells from human donors. HI assays were performed with recombinant SP-D preparations described above. Inhibition or disruption of agglutination was measured using serial dilutions of SP-D preparations.

##### Enzyme-linked Immunosorbent Assay (ELISA)

96-well plates were coated overnight at 4 °C with 0.1 μg/well of Phil-82, Phil-BS and PR-08. Serial dilutions of NCRDs were added to control wells. Wild-type huNCRD and mutant version D+R were used. Plates were washed 3x with 100 μl of PBS++ (PBS containing 0.9 mm CaCl_2_, 0.52 mm MgCl_2_ and 0.16 mm MgSO_4_) and blocked with blocking buffer (Superblock, Pierce). To the wells coated with IAV, different doses of NCRD, matching those in control wells, were added and incubated for 30 min at 37 °C. Plates were then washed 3× with wash buffer. To each well, horseradish peroxidase conjugated anti-S-protein antibody was added and incubated at 37 °C. Following incubation, plates were washed 3× with PBS++ and 50 μl of 1-step ELISA substrate (Pierce) was added. After 20 min incubation at room temperature with the ELISA substrate, the reaction was stopped by addition of 50 μl 1N sulfuric acid. Plates were read for absorption at 450 nm wavelength with wavelength correction at 540 nm.

##### Fluorescent Focus assay (IAV infectivity assays)

Infectivity assays were performed using human bronchial/tracheal epithelial (BTE) cells (Lifeline Cell Technology, Walkersville, MD). BTE cell monolayers were grown to about 70% confluency in 96 well plates. IAV preparations were pre-incubated with SP-D preparations for 30 min at 37 °C, to allow SP-D binding and inhibition of HA. The BTE cell monolayers were infected with these IAV preparations for 45 min at 37 °C, in PBS++. Cells were tested for presence of IAV after 18 h of virus addition using an anti IAV nucleoprotein monoclonal antibody (Millipore) and fluorescence detection, as described previously (56).

##### Surface Plasmon Resonance

Surface Plasmon Resonance (SPR) experiments were performed as described previously by Crouch *et al.* (53). Recombinant human SP-D NCRD (Neck and carbohydrate recognition domain) preparations were immobilized using N-terminal His tags on NiNTA, with the binding domain oriented upward. Binding of Phil/82 and Phil/82/BS B-HA were measured against recombinant wild type huNCRD and double mutant D+R.

##### Glycan Array Binding

Intact virions were labeled with Alexa 488 succinimidyl ester conjugated dye (Life Technologies/Thermo Fisher Scientific, Carlsbad, CA) using the manufacturer's protocol. Postlabeling, virions were dialyzed against 1× PBS. HA titers were measured and the virus preparations were appropriately diluted to adjust for differences in HA titers before binding experiments. Glycan array binding was performed using standard protocols at the Consortium for Functional Glycomics (57–59) Protein-Glycan Interaction Core at Emory University, using array version 5.2 (https://glycopattern.emory.edu/structures/view?version = 5.2). Binding was measured as fluorescent units of bound fluorescently labeled influenza virions.

##### Computational Glycobiology

##### 3D Structure Generation

A homology model for the Phil/2/1982 HA trimer was generated in Modeler (60–62) using PDBIDs 2YP3, 2YP7, 1HA0, 4KVN, 4WE5, 4O58, and 4WA1 as templates. In order to decrease the time required to perform a molecular dynamics (MD) simulation, a smaller structure was generated from the HA0 trimer that contained only residues F83 to D86 of the stalk and D57 to S270 of the head group. Glycans were attached to the head group using a customized version of the glycoprotein builder available on GLYCAM-Web (63), with the following modifications. When attaching the N-glycan to an Asn sidechain, the χ1, χ2, ψ, and ϕ torsion angles were adjusted to match observed rotamers reported for *N*-linked glycoproteins in the PDB (64). Any vdW overlaps were relieved by adjusting the glycosidic torsion angles within normal bounds (65). In all cases, the Asn sidechain conformation that was observed to be most common in the PDB was selected, provided that it could be attached without irreconcilable steric overlaps with the protein surface. Man_9_GlcNAc_2_ glycans were added to each of the five *N*-glycosylation sites on the HA headgroup; at sites Asn^63^, Asn^126^, Asn^144^, Asn^165^, and Asn^246^.

##### System Preparation for Simulation

hydrogen atoms were added to the head group structure, with the protonation states of the ionizable side chains being assigned by tleap (66). The structures were placed in a periodic box of ∼35,000 TIP5P waters (67) to provide an 8 Å buffer between the glycan and the edge of the periodic box.

##### Energy Minimization and Solvent Equilibration

energy minimization of all atoms was performed for 20,000 steps (10,000 steepest decent, followed by 10,000 conjugate gradient). The energy-minimized structures were equilibrated at 300 K under nPT conditions for 400 ps, with 5 kcal/mol-Å^2^ Cartesian restraints on the solute heavy atoms.

##### System Equilibration and Production MD

after solvent equilibration, the heavy atom restraints were removed, and 1 ns of equilibration MD followed by a 500 ns production simulation were performed. The Cα atoms of the terminus residues were restrained (5 kcal/mol-Å^2^).

##### Energy Minimization and Molecular Dynamics (MD) Simulations

all simulations were performed with the CUDA implementation of PMEMD (68, 69) in the Amber14 software suite (66). The GLYCAM06-j (63) and Amber14SB parameters (66) were employed for the carbohydrate and protein portions, respectively. A Berendsen barostat with a time constant of 1 ps was employed for pressure regulation, whereas a Langevin thermostat with a collision frequency of 2 ps^−1^ was employed for temperature regulation. A nonbonded interaction cutoff of 8 Å was employed. Long-range electrostatics were treated with the particle-mesh Ewald (PME) method (70). Covalent bonds involving hydrogen were constrained with the SHAKE algorithm allowing a time step of 2 fs (71).

##### Post Processing

An ensemble of 1000 conformations for the glycosylated Phil-2–1982 HA glycoprotein were taken at regular 500 ps intervals of the MD simulation using cpptraj (72).

##### Assessment of Glycan Accessibility to ERManI

A new crystal structure of ERManI, kindly provided by Dr. Kelley Moremen's laboratory, includes a full high mannose glycan in the binding site. This allowed assessment of the accessibility of the glycan to ERManI by superimposing the ERManI-bound Manβ1–4GlcNAcβ residues onto each *N*-glycan on the H3 head group. This was performed for each *N*-glycan over 1000 snapshots taken at regular intervals from the MD simulation. If surface overlaps greater than ∼36 Å^2^ (equivalent to a buried Carbon atom) were observed, the glycan was deemed to be inaccessible for that particular time point of the simulation.

##### Assessment of Glycan Accessibility to SP-D

A 3D structure has been determined previously for unliganded human SP-D (PDBID 1B08), as well as porcine SP-D in complex with Manα (PDBID 4DN8) (73). Despite structural differences in the carbohydrate binding sites of the porcine and human proteins, the relative spacing of the binding sites is identical, with a linear distance of ∼45 Å between each Manα binding site. By monitoring the distances between the non-reducing terminal Manα residues in each *N*-glycan over the course of the simulation, it was determined which sets of three *N*-glycans were positioned for trimeric interaction with SP-D (sets that were all 43–47 Å apart for any of the 1000 snapshots).

## RESULTS AND DISCUSSION

We modeled the changes in HA mature structure that occur as an H3N2 subtype evolves during repeated growth in eggs in the presence of bovine serum. This process rendered the virus resistant to conglutinin and mannose binding lectin in serum, and to SP-D as well (15). Owing to the error prone nature of virus replication and the inclusion of host proteins in the virion structure (74), we acquired deep proteomes of H3N2 before and after development of resistance to SP-D. We then profiled the range of glycans present on the virions and used this information to drive determination of confident site-specific glycosylation from glycoproteomics data. For HA, the combination of peptide proteolytic variants, other PTMs including oxidation and deamidation, and glycosylation yield an extremely large number of theoretical structures. To address this, we combined proteomics, glycomics and glycoproteomics analyses to produce site-specific glycosylation assignments.

We and others have developed liquid-chromatography-mass spectrometry methods with sufficient power for analysis of glycopeptides from complex glycoproteins (33, 75–78). In addition, improvements to software tools further enhance the ability to analyze complex glycoproteins (79, 80). We built an exhaustive glycopeptide bioinformatics search-space that made no assumptions regarding peptide backbone, proteolytic variants, PTMs, or glycome present in the IAV samples. We then defined the mature structures of H3N2 HA and the changes associated with escape from SP-D neutralization. We used viral infectivity assays, glycan array binding, lectin binding and other biochemical methods to demonstrate the changes in biological interactions and activities associated with evolution of glycosylation structure among IAV subtypes and strains. In order to understand how particular glycoforms are generated, we modeled the interactions between mature glycosylated HA and ER class I α-mannosidase (ERManI), a key enzyme in the glycan biosynthetic pathway. We then defined the key glycans mediating interactions of SP-D with HA using structure modeling and molecular simulation studies.

### 

#### 

##### Proteomics

Influenza A shows host species-specificity in infection governed by HA binding to sialylated glycans present on host cell-surface receptors. Avian-strains prefer binding to NeuAc-α-2,3-Gal, whereas human-strains prefer binding to NeuAc-α-2,6-Gal (81–84). Minor changes to the protein sequence correlate with a switch in sialic-acid binding preferences leading to changes in receptor specificities and altered susceptibility toward antibodies and lectins (17, 85–87).

We performed deep proteomics on deglycosylated IAV digests. Despite the fact that IAV contains only 8 gene segments, corresponding to roughly 11 proteins, the virions include host proteins as integral parts of its architecture (74). In addition, host proteins may copurify with the virions. We therefore accounted for all proteins and glycoproteins present in the samples in order to maximize the confidence of site-specific glycosylation assignments. The error-prone manner of IAV replication allows incorporation of mutations in viral proteins that may affect glycosylation sites and neighboring residues. Our deep proteomics analyses of virus preparations covered all glycosylation sites and identified glycopeptide variants that included other PTMs and incomplete proteolytic cleavages. We therefore used an unbiased bioinformatics search space for glycopeptide assignment.

In addition to identifying peptide backbone variants, we identified a revertant population in the Phil-BS sample. The *N*-glycosylation sequon at Asn^165^ apparently became disrupted because of an acquired mutation leading to conversion of NVTM to NVAM in the majority of Phil-BS virion population; however, a small fraction of the population contained the wild-type sequon NVTM. Based on extracted ion chromatogram peak areas, the revertant corresponded to ∼2% of the population (see Site occupancy analysis data). We observed no reversion at the second disrupted sequon (Asn^246^ NSTG) mutated in Phil-BS. To check for presence of glycosylation at the revertant sequon, we included both Phil-82 and Phil-BS protein sequences in the Phil-BS search hypothesis.

**Fig. 2.**
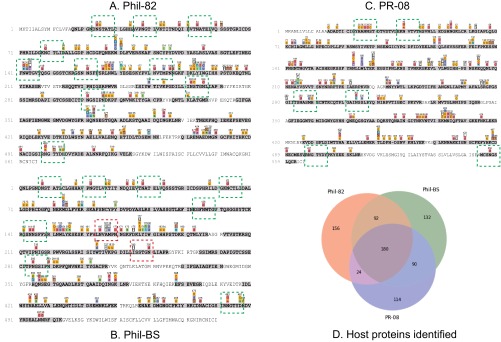
**Proteomics results.** Proteomics coverage of Phil-82 (*A*), Phil-BS (*B*) and PR-08 (*C*) hemagglutinins. Portions of sequence covered are highlighted in gray, with colored symbols above the sequence rows representing modifications and mutations. Green boxes show *N*-glycosylation sequons and red boxes indicate mutated/disrupted sequons in Phil-BS; D: Total number of proteins including host-proteins identified in proteomics of the three IAV samples. Only IAV and chicken (Gallus gallus) proteins identified with 2 or more unique peptides were counted.

A majority of proteins identified were common among the three IAV samples (Fig. 2*D*). Gene ontology analysis performed using STRAP (88), showed similar distributions of proteins in terms of molecular functions and cellular localization (see results in the Functional annotation of proteomics results). Functional annotation of the proteins showed close similarity to the results described by Hutchison *et al.* (74), showing a wide-variety of host-proteins involved in various structural and molecular roles present in the virions. We observed deamidation (NQ) as the most commonly encountered PTM in the proteomics results, followed by nonspecific carbamidomethylation, oxidation (M), sodium adduction and dehydration (supplemental Fig. S2).

We identified 10 and 8 sequons for Phil-82 and Phil-BS respectively, as shown in Fig. 2 and supplemental Fig. S1. All the peptide variants identified and used to construct our informed glycoproteomics bioinformatics hypotheses are shown in Protein and peptide sequences used in proteomics and glycoproteomics searches.

##### Glycan site-occupancy analysis

For modeling and molecular-dynamics studies, we determined glycosylation site occupancy of Phil-82 HA. We evaluated the NetNGlyc server (89) (http://www.cbs.dtu.dk/services/NetNGlyc/), used in published studies (90–93, 27) to provide a qualitative measure of the likelihood for *N*-linked glycosylation, given the protein sequence; however, we found that NetNGlyc predictions inconsistent with our results. Table I compares NetNGlyc predictions and site-occupancy results from proteomics data for Phil-82. Although NetNGlyc predicted no glycosylation at sites 38 and 144, we identified glycopeptides containing those two sites with high confidence from glycopeptide tandem MS data. The data showed low site occupancy at Asn^144^ and high occupancy for Asn^38^. These results re-emphasize the importance of analyzing both deglycosylated peptides and intact glycopeptides to minimize assumptions about samples and shifting reliance from prediction tools to empirical evidence.

**Table I:** NetNGlyc predictions for glycosylation site occupancy and site-occupancy from proteomics data for Phil-82

Site	NetNGlyc Potential	NetNGlyc Jury Agreement	NetNGlyc Results	Site-occupancy calculated from proteomics
8 NSTA	0.7987	9/9	+++	1.0
22 NGTL	0.7153	9/9	++	1.0
38 NATE	0.4996	3/9	-	0.99
63 NCTL	0.6366	9/9	++	0.99
126 NWTG	0.5393	6/9	+	0.73
144 NNSF	0.4293	6/9	-	0.17
165 NVTM	0.7626	9/9	+++	0.99
246 NSTG	0.5880	7/9	+	0.99
285 NGSI	0.6716	9/9	++	0.95
483 NGTY	0.5207	8/9	+	0.95

##### Glycomics

**Fig. 3.**
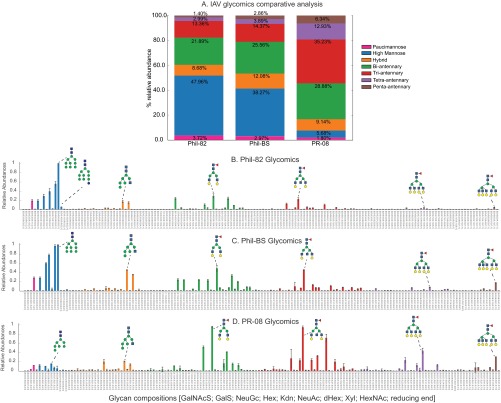
**Glycomics results from negative mode LC-MS profiling of released IAV *N*-glycans.** *A*, Comparison of the three strains. Stacked bars represent mean composite relative abundances of different glycan classes, categorized by HexNAc and Hexose units in the identified glycan compositions (Paucimannose: HexNAc = 2, Hexose < = 4; High Mannose: HexNAc = 2, Hexose > = 5; Hybrid: HexNAc = 3; Bi-antennary: HexNAc = 4; Tri-antennary: HexNAc = 5; Tetra-antennary: HexNAc = 6 and Penta-antennary: HexNAc = 7). Bar plots show individual glycan relative abundances for *B*, Phil-82; *C*, Phil-BS; *D*, PR-08. Colored bars represent mean abundances relative to the most abundant composition detected for each sample. Error bars represent standard deviation. The most abundant glycoforms in each category are labeled with putative topologies using the Consortium for Functional Glycomics glycan representation scheme.

We used glycomics profiling to define the range of glycans present on virions precisely. The glycan profiling results for Phil-BS (38% high-mannose) showed an ∼10% decrease in relative abundances of high-mannose type *N*-glycans compared with Phil-82 (48%) (Fig. 3*A*). PR-08 displayed a very small proportion of *N*-glycans (ca. 6%) occurring as the high mannose type. Detailed comparison of abundances for individual glycan compositions (Fig. 3*B*, 3*C*, 3*D*) showed a marked decrease in the relative abundances of immature Hex_9_HexNAc_2_ and Hex_10_HexNAc_2_ glycan compositions in Phil-BS. The glycan topologies shown in Fig. 3 are speculative. Glycomics profiling using LC-MS provides saccharide compositions only—topological/structural information cannot be derived from these experiments. Detailed results from glycomics analyses have been included in the data repository.

From the proteomics results, the primary difference between Phil-82 and Phil-BS was the disruption of two *N*-linked glycosylation sequons at positions 165 and 246. The glycomics results implied that a large proportion of the glycans occupying these two sequons were high-mannose type. To confirm this theory, we carried out intact glycopeptide analysis.

##### Glycoproteomics

**Fig. 4.**
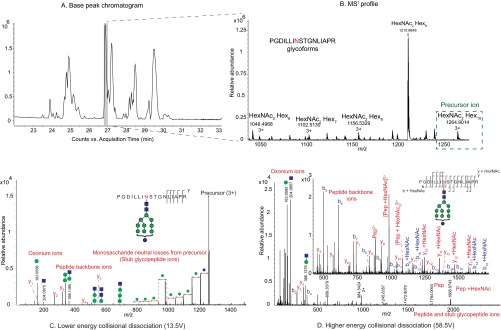
**Glycoproteomics analysis data dimensions.** *A*, Chromatographic separation and associated peak areas of enriched Phil-82 IAV glycopeptides; *B*, MS^1^ profiling and intact mass assignments of Phil-82 hemagglutinin glycopeptide PGDILLINSTGNLIAPR glycoforms; Tandem MS of glycopeptide PGDILLINSTGNLIAPR - Hex_10_HexNAc_2_ showing product ions resulting from lower-energy collisional dissociation (*C*) and higher-energy collisional dissociation (*D*). Glycan topologies shown are speculative based on inferred glycan compositions.

For confident assignment of site-specific glycoforms, we analyzed intact glycopeptides using LC-tandem-MS and mined the data using both naïve and informed bioinformatics search space hypotheses, as described in the experimental section. Collisional dissociation of glycopeptides in the gas-phase generates abundant ions from glycosidic bond dissociation; however, provided application of sufficient collision energy, peptide bond dissociation also occurs (33). Fig. 4 compares the features in collisional dissociation of glycopeptides using low *versus* high collision energies.

As seen in Fig. 4*C*, lower-energy collisional dissociation yields abundant ions from glycosidic bond dissociation. Higher collision energies (Fig. 4*D*) yield tandem mass spectra that contain glycan oxonium ions, peptide backbone dissociation ions and intact-peptide with glycan additions (known as stub-glycopeptides). We used these features in our automated data analysis workflow for glycopeptide assignment.

##### Integrated-omics

Analysis of intact glycopeptides is often challenging because of the complexity and size of the bioinformatics search space (33, 94). Typically, hypotheses for glycopeptide mass spectrometry data analysis are limited by assumptions about the peptide variants and glycoforms present in a sample. This limitation has been overcome in proteomics analyses with the use of error-tolerant database searches (95–98), which check for modifications (37) and mutations that best explain the data, in addition to those specified by the user. This is difficult to perform in case of glycopeptides because of the already large number of glycoforms associated with each glycosylation site. A number of software tools have emerged recently that mine only glycopeptide tandem MS (99–102) or perform both proteomics and glycoproteomics searches (103). However, the analyses are limited to user defined boundaries of proteoforms and glycoforms. Use of assumptions while defining these boundaries or search-spaces can lead to under or over-estimation of the proteoforms, thus leading to loss of information or high false discovery rates, respectively.

For a glycoprotein as complex as HA, tandem MS can be used to resolve ambiguous assignments; however, glycopeptide tandem MS alone does not allow adequate depth and confidence in assignment for glycoproteoforms. Additionally, glycoproteomics alone does not account for possible mutations incorporated into viral genome as a result of evolutionary pressures.

To produce the most confident information on influenza glycoproteome, we adhered to a *lex parsimoniae* approach, described here as “integrated-omics.” Proteomics and glycomics workflows are well-established and easy to implement. As a first step in the workflow, we performed bottom-up proteomics on PNGase*F* deglycosylated influenza virions. The data were mined using hybrid *de novo* and error-tolerant proteomics database searches, which accounted for all possible PTMs, mutations and peptide variants from sample handling. The released IAV *N*-glycans were analyzed in a separate experiment and searched against glycan databases to define the glycoform search space. Thus, in an effort to minimize assumptions and efficiently mine glycoproteomics data, the glycomics and proteomics information were integrated to generate a glycopeptide database, against which the glycopeptide MS and tandem MS data were searched. Tandem MS matches were scored based on fragment ion coverage. To calculate a confidence score, a decoy database containing reversed target sequences was used, where the three residue sequon was maintained in the correct order. For example, glycopeptide SVQEIQEIQTFFYFTPN(HexNAc)KTEDTIFLR had a decoy LFITDEN(HexNAc)KTPTFYFFTQIEQIEQVSR. Confidence scores (q-values) were calculated as described by Käll *et al.* (46, 104).

The tandem mass spectrometric assignment scores for some glycopeptides, such as those spanning site 126 (NWTG) were lower than the accepted score threshold, because of incomplete coverage and low precursor and product ion abundances. Manual validation helped confirm the assignments and inclusion in the final results. All automated glycopeptide assignment results have been included as comma separated text files in the data repository.

**Fig. 5.**
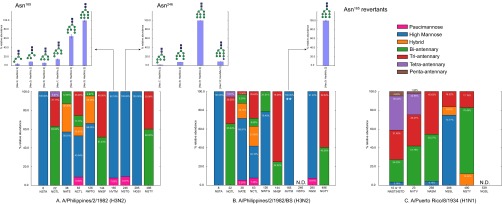
**Integrated-omics results.** Site-specific glycan distributions for hemagglutinins. *A*, Phil-82; *B*, Phil-BS; *C*, PR-08. Stacked bars represent composite mean relative abundances for *N*-glycan compositions categorized by number of HexNAc units, as described above. All stacked bars have been scaled to 100% to represent percentages of individual glycan classes among all glycans identified. Insets show individual glycoform relative abundances for sites identified in the interaction with immune-lectins. Error bars represent standard deviation in three measurements of glycoform abundances. N.D.: Not Detected. **Revertant population at site 165 in Phil-BS.

The site-specific glycosylation plots, shown in Fig. 5, represent normalized percent relative abundances of different glycoform classes at each glycosylation site for the hemagglutinins from three IAV strains. Although the plots present results from glycopeptide LC-tandem MS analyses, the databases against which glycopeptide LC-tandem MS data were searched were generated by combining the proteomics and glycomics results for respective virions. These results therefore reflect the integration of different data domains. Individual glycan compositions contributing to the abundances at sites 165 and 246 have been shown in Fig. 5 (inset). It is clear from the integrated-omics results that glycosylation at Asn^165^ and Asn^246^ is lost in the escape mutant Phil-BS from the selection pressures inflicted by growth in presence of bovine serum. All site-specific glycan distributions are shown in supplemental Fig. S3, and aligned to individual HA monomer models for better visualization in supplemental Fig. S4. Supplemental Fig. S5 compares glycan distributions from glycomics results with those from integrated-omics results. The figures show good correlation between relative abundances for high-mannose type glycans (2 HexNAc units) and hybrid/complex type glycans (>2 HexNAc units) from glycoproteomics and glycomics data. LC-MS profiling based glycomics is error-prone because multiple glycan compositions can match the given mass and also because noise peaks can sometimes get assigned to glycan compositions. Thus, glycopeptide tandem-MS becomes an essential step in the integrated-omics workflow, to eliminate false-positives and generate confident site-specific glycan profiles.

Hartley *et al.* concluded that Asn^246^ on Phil-82 and Phil-BS is not occupied by a glycan (24). These conclusions were derived using results from genetic sequence analyses and mobility shifts in electrophoresis, showing loss of glycosylation in escape mutants. By contrast, we observed that Asn^246^ is in fact glycosylated in Phil-82, whereas Phil-BS loses glycosylation at both Asn^165^ and Asn^246^, because of acquired mutations. This exemplifies the need for site-specific structural information on glycoproteins, which genetic sequencing and classical biochemical methods fail to provide.

Consistent with the proteomics results, we identified glycopeptides from a revertant population only for Asn^165^ (NVTM); we observed no reversion at Asn^246^ (NSTG). To check what percentage of the total virus population reverted to the intact sequon at Asn^165^, we compared arbitrary unit counts from integrated extracted ion chromatograms in the proteomics data. We also calculated site-occupancy for this revertant population as described above. In the proteomics analyses, 2% of the total Phil-BS population contained the intact revertant sequon (NVTM) at Asn^165^, with a glycan site-occupancy of 92%.

Glycosylation changes were limited to loss of glycosylation at Asn^165^ and Asn^246^, whereas that at other sequons remained largely unchanged. Both Asn^165^ and Asn^246^, present on the HA head, are accessible to host immune lectins. Any differences observed in bioassays could therefore be attributed to differences in glycosylation at these two sites. Although, high-mannose glycans dominated at all sites on H3 HA head, the level of glycan processing varied. In particular, for Phil-82, we noticed a striking difference in extent of high mannose *N*-glycan processing between Asn^165^ and Asn^246^. In the early stages of biosynthesis, glycoproteins are occupied by glucosylated high mannose (Glc_3_Man_9_GlcNAc_2_) *N*-glycans. The glucose residues are recognized by calnexin and calreticulin as a quality-control mechanism for protein folding. The three glucose units are removed subsequently in the endoplasmic reticulum by glucosidases. A significant number of glycoproteins are also acted upon by ER mannosidase-I, which removes a terminal mannose unit, after which the glycoproteins are transported to the Golgi apparatus with a Man_9_GlcNAc_2_ or Man_8_GlcNAc_2_ glycan. In the Golgi apparatus, the mannose residues are trimmed further before becoming extended into complex type structures (105–111). Both Asn^165^ and Asn^246^ displayed higher abundances of relatively immature glycans containing a higher number of hexose units in their compositions, as seen in the site-specific glycan distributions (Fig. 5 inset and supplemental Fig. S3). The composition Hex_9_HexNAc_2_ was the most abundant at both Asn^165^ and Asn^246^ and the presence of Hex_10_HexNAc_2_ was observed at Asn^246^ by tandem MS, as shown in Fig. 4 (*C* and *D*).

The glycosylation profile on PR-08 contrasted with those of the two H3N2 strains in that complex type *N*-glycans dominated at all sequons. Given that all three viruses were grown using the same egg culture conditions, this ruled out the involvement of external factors including cell type, growth media and culture system in presence of under-processed *N*-glycans on H3N2 virions. Another recent study has shown similar predominance of high-mannose type glycans on the head region of a different H3N2 strain (27). Although the homologous H3 HAs of the same subtype but different strains show similar glycosylation profiles, the H1 HA from PR-08 contains much more highly processed *N*-glycans. This implicates protein backbone and structural features of the viral subtypes and strains as the drivers in glycan processing and resulting microheterogeneity.

##### Bioassays

**Fig. 6.**
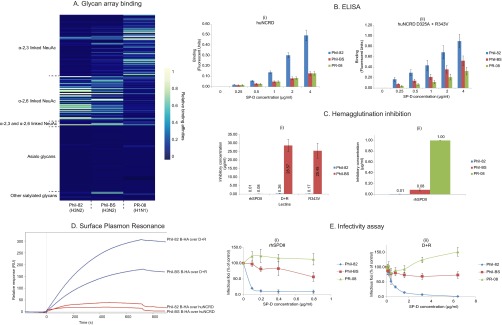
**Results from biochemical, virological and immunological assays.** *A*, CFG glycan array analysis of three IAV strains. Only glycans where relative binding was greater than 0.1% for any one of the three strains have been included in the heat-map. Array glycans are sorted into different categories (y-axis) and the three virus strains are listed on x-axis; *B*, ELISA results. Bar-charts show dose-dependent binding to human surfactant protein-D NCRD wild-type (i) and double mutant D+R (ii). X-axis shows the concentration of SP-D used and Y-axis shows binding response in fluorescent units measured. Error bars represent standard error; *C*, Hemagglutination inhibition of IAV strains. (i) Comparison of inhibitory effect of different lectins on Phil-82 and Phil-BS. (ii) HA inhibitory concentrations of rhSPDII for the three IAV strains studied. Error bars represent standard error; *D*, Surface Plasmon Resonance results. Bromelain cleaved hemagglutinin (B-HA) from Phil-82 and Phil-BS, were introduced in the mobile phase and binding toward immobilized wild type or mutant NCRDs was measured; *E*, Infectivity assay results. Infectivity of the three IAV strains was compared after pre-incubation with increasing concentrations of (i) rhSPDII and (ii) D+R mutant SP-D. Data points show infectivity measured as infectious foci expressing influenza nucleoprotein, relative to control (no SP-D pre-incubation).

We used biochemical and virological assays to survey virion binding preferences to host glycans and correlate differences in glycosylation with susceptibility of HAs toward the immune lectin SP-D (Fig. 6).

##### Glycan Array Binding

In addition to the genetic reassortments that cause antigenic-shift and zoonosis, differences in protein backbone and in glycosylation states affect receptor binding specificities of influenza A virus (87, 112–117). Glycan array results, in Fig. 6*A*, show binding affinities of the three fluorescently labeled IAV strains to glycans normalized to the strongest binder for each strain. Only glycans where the relative binding was found greater than 0.1% of the strongest bound glycan for any IAV strain have been included. A complete list of glycans and fluorescence readouts from bound IAV are included in Glycan array binding data/results.

Both Phil-82 and Phil-BS strains showed preferential binding to α-2,6 linked sialic acid containing glycans, *versus* α-2,3 linked for the PR-08 H1N1 subtype (Fig. 6A). This showed that the reassorted H3N2 virions, generated with A/Puerto Rico/8/34 backbone, retain the binding specificities on their HAs as expected. Furthermore, the mutations induced by selection pressure leading to loss of two *N*-linked glycans on Phil-BS, did not alter its binding preferences for sialylated glycans and therefore host specificity.

##### ELISA

ELISA results performed at different concentrations of the wild-type huNCRD show stronger binding for Phil-82, compared with Phil-BS and PR-08, which show similar binding affinities, as seen in Fig. 6*B*(i). The D+R mutant huNCRD has mutations in the amino acids surrounding the lectin site that improve hemagglutination activity (48, 118). Consistent with previous reports, we observed higher binding for all three strains with D+R mutant huNCRD Fig. 6*B*(ii), the extent of which was substantially higher in the case of Phil-82 and Phil-BS than for PR-08. These results correlated well with our observed abundances for high-mannose type glycans present on the three strains from integrated omics. Although Phil-BS had lower abundance of high-mannose type glycans compared with Phil-82 because of loss of glycosylation at sites 165 and 246, the abundances of immature glycans remained higher than on PR-08, for which mature complex type *N*-glycans predominated.

##### Hemagglutination Inhibition

Surfactant protein-D plays an important role in inhibition of IAV as part of the host innate immune response. SP-D has a carbohydrate recognition domain that binds to mannose containing *N*-glycans on viral hemagglutinin. This presumably inhibits HA binding to host cell surface receptors, preventing docking and entry in to the host cell. Typical hemagglutination inhibition assays are performed using serum to detect the presence and levels of antibodies against HA. Because SP-D prevents IAV interaction with host cells, it can be used to inhibit hemagglutination activity, in a dose-dependent manner(15, 20, 54).

We first determined the sensitivity of Phil-82 and Phil-BS strains toward SP-D lectins. As seen in Fig. 6*C*(i), rhSPDII (full-length SP-D) was the most effective in inhibiting the hemagglutination activity between the three SP-D constructs compared. Although rhSPDII inhibited both Phil-82 and Phil-BS, the D+R and R343V constructs were effective only against Phil-82. Fig. 6*C*(ii) shows the measured inhibitory concentrations of rhSPDII for all three IAV strains. Phil-BS required eightfold higher rhSPDII concentration than Phil-82, whereas PR-08 could not be inhibited even at the highest lectin concentration (1 μg/ml), used in the experiment. PR-08 was also found to be insensitive to D+R and R343V (data not shown). We concluded from these ELISA and hemagglutination inhibition assay results that the presence of high-mannose type *N*-glycans on the virus surface correlates with extent of neutralization of hemagglutination by SP-D.

##### Surface Plasmon Resonance

Although HA is almost 10 times more abundant than NA (74), it was important to identify the primary contributor to SP-D interactions and changes therein. We therefore performed SPR experiments using bromelain cleaved hemagglutinins (B-HA) from the wild-type and mutant H3N2 IAV strains. As shown in Fig. 6*D*, both wild-type and mutant SP-Ds display markedly stronger binding to the Phil-82 B-HA compared with Phil-BS B-HA. Thus, differing SP-D binding among the tested IAV strains can be attributed to changes in the HA. Therefore, ELISA, hemagglutination inhibition and SPR assays established a clear difference in SP-D binding and activity of HA from different IAV strains.

##### Infectivity Assay

We next determined the ability of SP-D to neutralize virus infectivity using fluorescent focus assays. For these experiments we used primary human bronchial/tracheal epithelial cells to ensure that the results reflected behavior of the viruses in non-malignant human respiratory epithelia. Infectivity was measured via production of viral nucleoprotein by infected host cells, using an anti-nucleoprotein antibody. Cells were infected using virions pre-incubated without SP-D or with different concentrations of full-length rhSPDII and the strong binding NCRD mutant D+R. Fig. 6*E* shows infectivity of the three virus preparations relative to cells infected without SP-D pre-incubation. With increasing concentration of rhSPDII during pre-incubation, the infectivity of Phil-82 declined sharply. By contrast, Phil-BS showed only a slight decrease in infectivity at higher lectin concentrations. With trimeric NCRD D+R, Phil-82 required pre-incubation with higher concentrations of lectin for loss in infectivity whereas no significant loss in infectivity was observed for Phil-BS. PR-08 infectivity was insensitive to either lectin preparation.

These results substantiate the involvement of Asn^165^ and Asn^246^ in HA interactions with SP-D, a collectin that attenuates viral interactions with host cell surface receptors and causes viral aggregation and recognition by immune cells. We concluded that the presence of high-mannose type glycans at Asn^165^ and Asn^246^ is crucial toward recognition by collectins.

##### Modeling and Molecular Dynamics

The integrated-omics results and bioassays showed reduced glycan processing at the two sites involved in immune recognition for Phil-82. The predominance of the Hex_9_HexNAc_2_ composition at these sites, suggested decreased processing by ERManI. The presence of Hex_10_HexNAc_2_ at Asn^246^ also suggested a decreased degree of *N*-glycan processing by other enzymes, including glucosidases. We therefore performed three-dimensional (3D) structural modeling and molecular dynamics (MD) simulations to understand the structural basis for the low level of glycan processing at these sites and the interactions of these immature glycans with SP-D.

##### Molecular Dynamics Simulation

A 500 ns simulation was performed on the head domain of the H3 trimer, with Man_9_GlcNAc_2_ attached to each of the 15 glycosites (namely Asn^65^, Asn^144^, Asn^126^, Asn^165^, and Asn^246^ on each protomer). Despite the initial threefold symmetry of the trimer, unique glycan-glycan and glycan-protein interactions formed on different protomers, a consequence of both the algorithm employed to attach the glycans and of the randomly assigned initial atomic velocities. Over the timescale of the simulation the unique interactions observed on individual protomers were stable and did not interconvert, indicating that interconversion occurs on larger timescales.

##### Assessment of Glycan Accessibility to ERManI

**Fig. 7.**
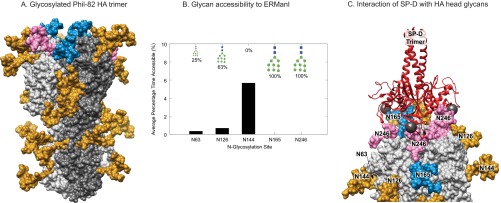
**Structure modeling and molecular dynamics results.** *A*, 3D model of glycosylated Phil-82 hemagglutinin trimer. One HA monomer is shown in dark gray. Glycans at Asn^165^ and Asn^246^ are shown in blue and pink, respectively. *B*, Percentage of simulation time that an *N*-glycan is accessible to ERManI. The values are averaged over each protomer of the H3 trimer. The amount of Man_9_GlcNAc_2_ at each site is depicted on the upper axis, demonstrating that increased accessibility to ERManI correlates with less Man_9_GlcNAc_2_ at each site. *C*, A model of trimeric SP-D (red ribbons) bound to three *N*-glycans on the H3 head domain (light gray surface). The 3D structure of the glycosylated H3 was taken from the MD simulation. In this snapshot, Manα residues at sites Asn^246^, Asn^165^, and Asn^246^ on different protomers were ∼45 Å apart, such that SP-D could bind each residue simultaneously. The calcium atoms found in each SP-D Manα binding site are shown (dark gray spheres).

The accessibility of each *N*-glycan to ERManI was assessed over the course of the simulation. The co-complexed *N*-glycan in the ERManI 3D structure was superimposed onto each N-glycan on the H3 head domain. Large surface overlaps between ERManI and the H3 glycoprotein indicated that the *N*-glycan was inaccessible at that particular time point of the simulation. The accessibility varied depending on the unique structures formed by the glycans on each protomer, but the relative percentage of time an *N*-glycan was accessible to ERManI correlated well with the amount of processing observed experimentally (Fig. 7*B*).

Both Asn^165^ and Asn^246^ remained completely inaccessible to ERManI throughout the simulation, which agrees with the experimental observation that these sites contain only high mannose glycans. Glycans at Asn^63^ and Asn^126^ which showed some processing to mature glycoforms, become accessible to ERManI over the course of the simulation. The most processed site, Asn^144^, adopts shapes accessible to ERManI for the longest amount of time during the simulation.

These results demonstrate a clear correlation between the percentage of time an *N*-glycan is accessible to ERManI and the degree of processing in the glycoforms observed at a site; however, the results indicate that longer simulation times, not feasible using present state-of-the art processors, would be required to observe interconversion between the conformations adopted at each site. Further, it should be noted that the actual value of percentage time available is based on an arbitrary overlap cutoff, and that assessment of accessibility does not account for induced fit in ERManI or any of the overlapping glycans and protein. Thus it is appropriate to consider only the relative values among sites.

SP-D binding to glycosylated HA - SP-D binds to Manα, which is present in high-mannose *N*-glycans that coat viral surface proteins. As SP-D adopts a trimeric structure with ∼45 Å between each binding site, we hypothesized that sets of *N*-glycans on the H3 head domain may be positioned such that SP-D could form a multimeric interaction with three *N*-glycans simultaneously, enhancing the interaction strength (see Fig. 7*C*). It is interesting to note that HAs also have 3-fold symmetry and a ∼45 Å linear distance between binding sites.

We took an ensemble of conformations adopted by the Man_9_GlcNAc_2_ glycosylated H3 from the MD simulation. By monitoring the distances between the non-reducing terminal Manα residues in each *N*-glycan over the course of the simulation, we determined which sets of three *N*-glycans were positioned for trimeric interaction with SP-D (sets that were 43–47 Å apart). Of the 20 total sets, 18 contained either Asn^165^ or Asn^246^ (Table II). The data imply that removal of Asn^165^ and Asn^246^ would greatly reduce the potential for trimeric interactions to form between SP-D and the H3 head domain. This finding agrees with the experimental assays (Fig. 6), where we observed that removal of these two glycosites reduced binding of SP-D.

**Table II:** Sets of N-glycosylation sites^a^ whose spacing and glycan dynamics allow trimeric interaction with a trimeric SP-D. ^a^Asn^144^ was excluded from the analysis as it was found to have both low site occupancy and a mature glycoform distribution

Sets of N-glycan sites (protomer number)		
Site 1	Site 2	Site 3
Asn^165^(1)	Asn^126^(1)	Asn^63^(3)
	Asn^246^(1)	Asn^126^(3)
Asn^165^(2)	Asn^165^(3)	Asn^63^(1)
	Asn^63^(2)	Asn^126^(3)
Asn^165^(3)	Asn^63^(1)	Asn^126^(2)
Asn^246^(1)	Asn^126^(2)	Asn^126^(3)
	Asn^165^(2)	Asn^126^(3)
		Asn^165^(3)
		Asn^246^(3)
	Asn^165^(3)	Asn^126^(2)
		Asn^126^(3)
	Asn^246^(2)	Asn^126^(1)
	Asn^63^(1)	Asn^126^(2)
Asn^246^(2)	Asn^126^(2)	Asn^165^(3)
		Asn^246^(3)
		Asn^63^(1)
Asn^246^(3)	Asn^126^(2)	Asn^63^(1)
		Asn^63^(2)
Asn^126^(2)	Asn^63^(1)	Asn^63^(2)
	Asn^63^(2)	Asn^126^(3)

We observed that the binding sites of trimeric architecture of SP-D are positioned perfectly to interact with glycans on the exposed influenza H3 head domain. Our molecular modeling predicts that the high mannose *N*-glycan at sites Asn^165^ and Asn^246^ play a pivotal role in forming trimeric interactions with SP-D, in agreement with the experimental evidence.

Enzyme accessibility is only one of the factors that determine the degree of glycan processing at a particular site. Residues outside of the traditional N-X-S/T (where X ≠ P) sequon can profoundly affect both the rate of glycosylation by oligosaccharyltransferase and the predominant glycoform observed (119). Further, mechanisms that govern intracellular proteostasis have been observed to impact the *N*-glycosylation pattern of secreted glycoproteins (120). We note, however, that a clear correlation between the relative accessibility of an *N*-glycan computed from an MD simulation and the degree of processing has been observed both here and in previous work (111).

##### Concluding Remarks

Several recent studies have underlined the importance of HA glycosylation in influenza virus pathology (5, 114, 121–124). In addition, a number of groups have attempted to trigger a broadly neutralizing antibody response toward influenza hemagglutinins, by artificially modulating HA glycosylation (125, 126). Therefore, HA glycosylation is a clear indicator of virus fitness and evolution (11, 13, 21, 122). Naturally, the ability to predict the fitness of an IAV strain based on its glycosylation profile will help designate strains for vaccine development.

We identified trends in viral evolution with a focus on changes in glycosylation patterns. Our results detailed the site-specific structural changes in HA glycosylation that occur as IAV adapts to immune pressure. We showed that not only the presence or absence of glycosylation, but also the level of glycan processing need to be considered with regard to prediction of interaction with molecules of the host immune system.

Most importantly, we demonstrated how comprehensive analysis of site-specific glycosylation fills in the gaps between modeling and structural studies. We demonstrated that with detailed site-specific glycosylation information, and computational glycosylation modeling, we can identify structural determinants of site-specific glycoforms. Our study describes a new strategy to elucidate IAV-host interactions and controlled evolution studies for understanding the effects of selection pressures in virus evolution.

## Supplementary Material

Supplemental Data

## Footnotes

^1^ The abbreviations used are:

IAV: Influenza A virus
CAD: Collision-activated dissociation
D+R: NCRD with two amino acid mutations
ERManI: Endoplasmic reticulum class I α-mannosidase
HA: Hemagglutinin
HCD: Higher-energy collisional dissociation
HILIC: Hydrophilic-interaction liquid chromatography
huNCRD: Wild-type human neck and carbohydrate recognition domain of SP-D
LC-MS: Liquid chromatography-mass spectrometry
MD: Molecular Dynamics
MS/MS: Tandem mass spectrometry
MS^2^: Tandem mass spectrometry
Phil-82: A/Philippines/2/1982 (H3N2)
Phil-BS: A/Philippines/2/1982/BS (H3N2) (Bovine serum escape mutant)
PR-08: A/Puerto Rico/8/1934 (H1N1)
PTM: Post-translational modification
R343V: NCRD with single amino acid mutation
rhSPDII: Recombinant full length human SP-D
SP-D: Surfactant protein-D.
