# Supplementary material for: Integrated Omics and Computational Glycobiology Reveal Structural Basis for Influenza A Virus Glycan Microheterogeneity and Host Interactions
Source: Mol Cell Proteomics. 2016 Mar 16;15(6):1895–912. doi: 10.1074/mcp.M116.058016 (PMC5083086; doi:10.1074/mcp.M116.058016)
Supplement: Supplemental Data [file supp_15_6_1895__index.html]

Integrated omics and computational glycobiology reveal structural basis for Influenza A virus glycan microheterogeneity and host interactions — Integrated Omics and Computational Glycobiology Reveal Structural Basis for Influenza A Virus Glycan Microheterogeneity and Host Interactions — Integrated Omics of Influenza A Virus Hemagglutinin — Supplemental Data 

# Integrated Omics and Computational Glycobiology Reveal Structural Basis for Influenza A Virus Glycan Microheterogeneity and Host Interactions

## Supplemental Data

- Site occupancy analysis (.zip, 1.5 MB) - Site occupancy analysis data.
- Functional annotation of proteomics results (.zip, 132 KB) - Functional annotation of proteomics results
- Glycan array binding data/results (.zip, 231 KB) - Glycan array binding data/results
- Protein and peptide sequences used in proteomics and glycoproteomics searches (.zip, 565 KB) - Protein and peptide sequences used in proteomics and glycoproteomics searches
- Calculated averages and errors from replicate glycomics measurements (.zip, 17 KB) - Calculated averages and errors from replicate glycomics measurements
- Supplemental Data (.pdf, 1.7 MB)
- Supplemental Data (.pdf, 1.7 MB)
