# Supplementary material for: Integrated Omics and Computational Glycobiology Reveal Structural Basis for Influenza A Virus Glycan Microheterogeneity and Host Interactions
Source: Mol Cell Proteomics. 2016 Mar 16;15(6):1895–912. doi: 10.1074/mcp.M116.058016 (PMC5083086; doi:10.1074/mcp.M116.058016)
Supplement: Supplemental Data [file 10.1074_M116.058016_mcp.M116.058016-6.pdf]

Supplemental information

Proteomics results:

Raw data, mzIdentML results and peaklists (mgf) have been supplied as supplemental files for all the proteomics results. File names indicate protease used and whether or not samples were deglycosylated using PNGaseF before analysis. The files are available on the following PRIDE repository:

Project Name: Influenza A virus- integrated glycomics, proteomics and glycoproteomics

Project accession: PXD003498

Project DOI: 10.6019/PXD003498

Keys to data:

Tryp- tryptic digest

Chymo- Chymotryptic digest

O16- Samples subjected to deglycosylation in presence of regular water ($H_2{}^{16}O$).

GP- No deglycosylation.

Glycomics results:

Raw data and GlycReSoft MS1 matching outputs have been included for all glycomics samples. MS1 scores are calculated as described previously (1).

Glycoproteomics results:

Results from glycopeptide data searched against database generated using proteomics results; along with raw LC-MS/MS data have been included in the PRIDE repository. The repository also contains annotated spectra for all glycopeptide spectrum matches.

MS2 scores are representative of number of fragment ions found (peptide backbone and stub ion coverage) (2). q-values depict minimal FDR threshold at which the identification is accepted as described by Käll et al. (3).

Functional annotation of proteomics results:

Proteins identified from proteomics analyses of tryptic $H_2{}^{16}O$ deglycosylated samples were subjected to gene ontology/functional annotation using STRAP (4). The results have been provided as part of the supplemental material (supplemental file 2), which can be opened and viewed using STRAP. http://www.bumc.bu.edu/cardiovascularproteomics/cpctools/strap/

The following criteria were used for exporting proteins for functional annotation:

-10lgP Peptides >= 15

-10lgP Proteins >= 20 and >= 2 unique peptides

De novo only ALC (%) = 50

Site-occupancy analysis data:

Site-occupancy analysis results for Phil-82 including integrated extracted-ion chromatograms have been included as an excel file (supplemental file 1).

Protein and peptide sequences:

Protein sequences for each of the three hemagglutinins and the glycopeptide compositions against which the data were searched have been provided as fasta files (*.fa) as part of the supplemental information (supplemental file 4).

Glycan array binding results:

Glycan array binding results from the Consortium for Functional Glycomics have been provided in supplemental file 3.

Glycomics results with means and errors:

As per journal guidelines, average values and standard deviations from replicate measurements in glycomics experiments have been provided in supplemental file 5.

Proteomics database:

The combined Uniprot and IAV database against which the proteomics data were searched has also been provided in a fasta format in the PRIDE repository.

Source code:

Source code for the automated data analysis workflow is hosted on: https://github.com/GlycReSoft2

Supplemental Figure 1:

Higher resolution figures for proteomics results (Figure 2 in main text):

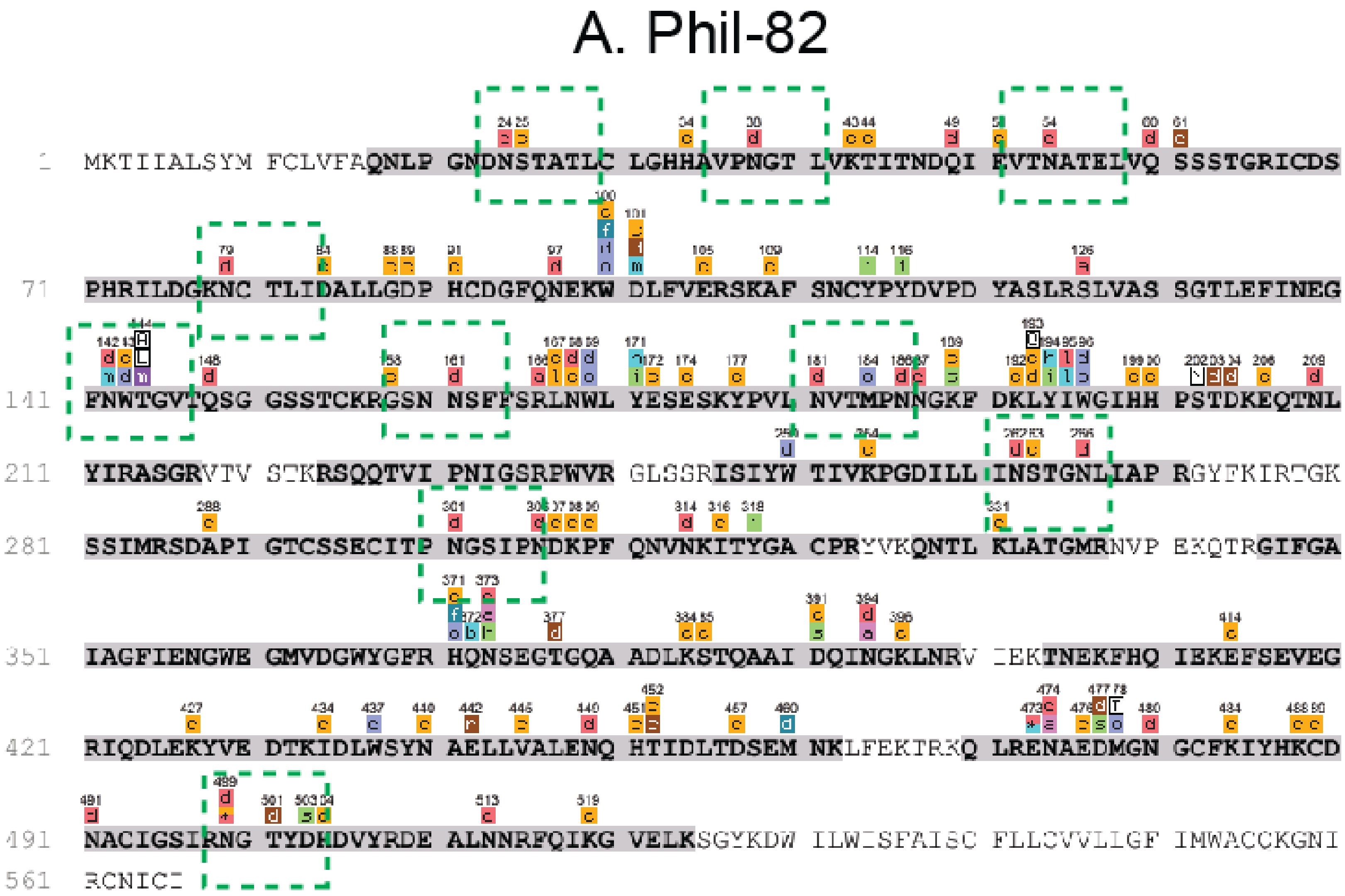

B. Phil-BS

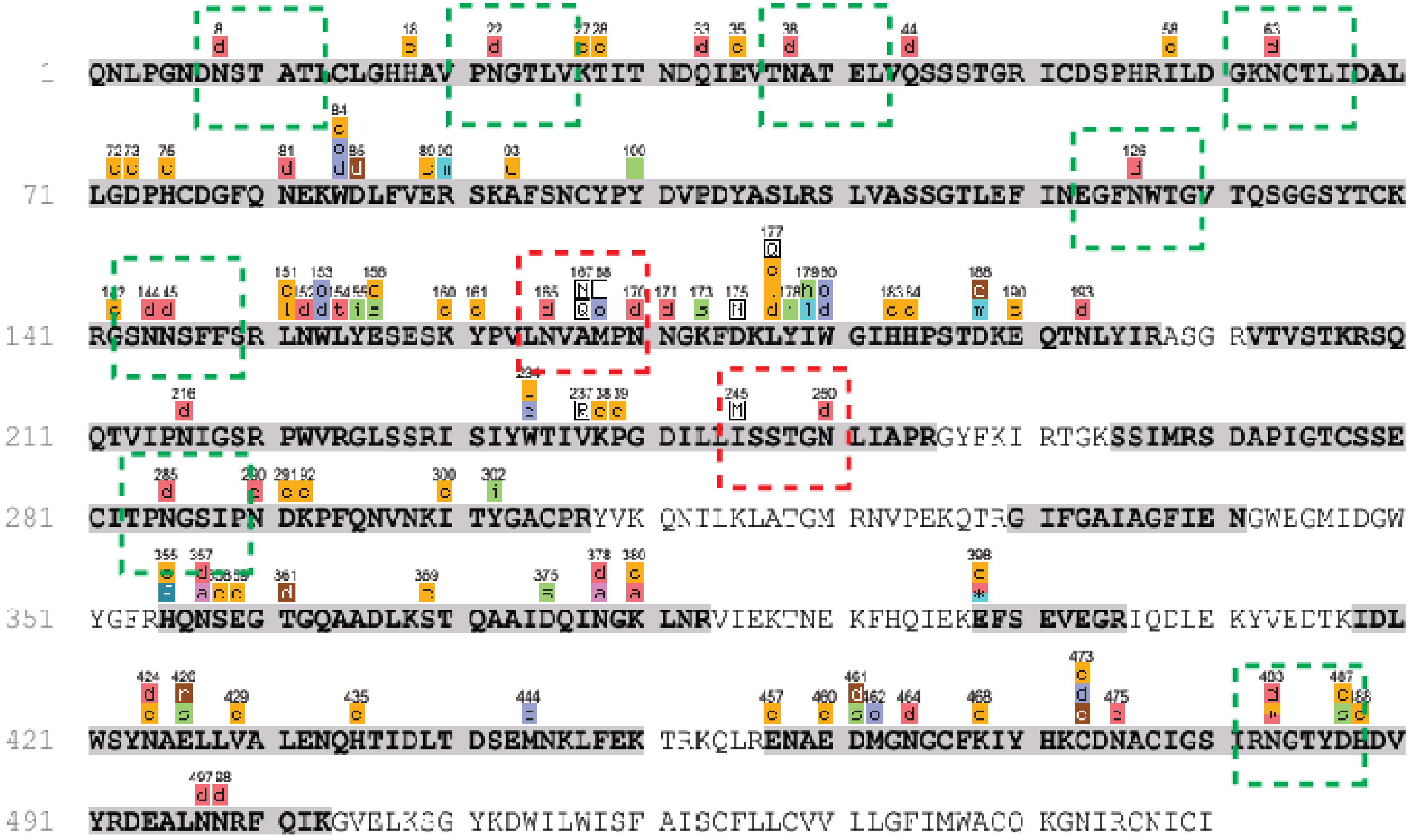

1 QNLPGNDNST ATLCLGHHAV PNGTLVKTIT NDQIEVTNAT ELVQSSSTGR ICDSPHRILD GKNCTLIDAL
71 LGDPHCDGFQ NEKWDLFVER SKAFSNCYPY DVPDYASLRS LVASSGTLEF INEGFNWTGV TQSGGSYTCK
141 RGSNNSFFSR LNWLYESESK YPVLNVAMPN NGKFDKLYIW GIHHPSTDKE QTNLYIRASG RVTVSTKRSQ
211 QTVIPNIGSR PWVRGLSSRI SIYWTIVKPG DILLISSTGN LIAPRGYFKI RTGKSSIMRS DAPIGTCSSE
281 CITPNGSIPN DKPFQNVNKI TYGACPRYVK QNTLKLATGM RNVPEKQTRG IFGAIAGFIE NGWEGMIDGW
351 YGFRHQNSEG TGQAADLKST QAAIDQINGK LNRVIEKTNE KFHQIEKEFS EVEGRIQDLE KYVEDTKIDL
421 WSYNAELLVA LENQHTIDLT DSEMNKLFEK TRKQLRENAE DMGNGCFKIY HKCDNACIGS IRNGTYDHDV
491 YRDEALNNRF QIKGVELKSG YKDWILWISF AISCFLLCVV LLGFIMWACQ KGNIRCNICI

C. PR-08

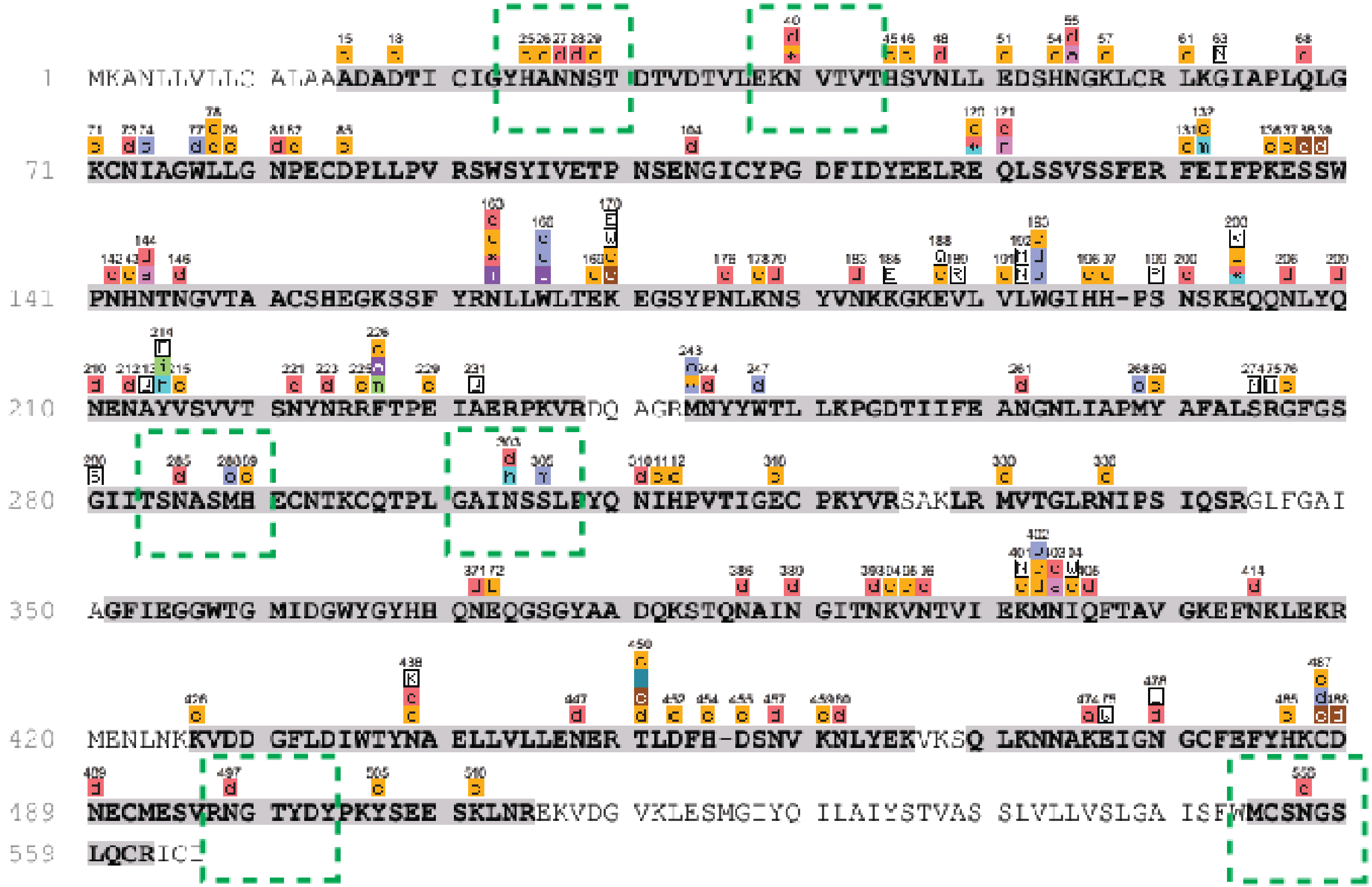

1 MKANLLVLLC ALAAADADTI CIGYHANNST DTVDTVLEKN VTVTHSVNLL EDSHNGKLCR LKGIAPLQLG
71 KCNIAGWLLG NPECDPLLPV RSWSYIVETP NSENGICYPG DFIDYEELRE QLSSVSSFER FEIFPKESSW
141 PNHNTNGVTA ACSHEGKSSF YRNLLWLTEK EGSYPNLKNS YVNKKGKEVL VLWGIHH-PS NSKEQQNLYQ
210 NENAYVSVVT SNYNRRFTPE IAERPKVRDQ AGRMNYYWTL LKPGDTIIFE ANGNLIAPMY AFALSRGFGS
280 GIITSNASMH ECNTKCQTPL GAINSSLPYQ NIHPVTIGEC PKYVRSAKLR MVTGLRNIPS IQSRGLFGAI
350 AGFIEGGWTG MIDGWYGYHH QNEQGSGYAA DQKSTQNAIN GITNKVNTVI EKMNIQFTAV GKEFNKLEKR
420 MENLNKKVDD GFLDIWTYNA ELLVLLENER TLDFH-DSNV KNLYEKVKSQ LKNNAKEIGN GCFEFYHKCD
489 NECMESVRNG TYDYPKYSEE SKLNREKVDG VKLESMGIYQ ILAIYSTVAS SLVLLVSLGA ISFWMCSNGS
559 LQCRICI

Supplemental Figure 2:

PTM frequencies:

The plot below shows the frequencies (as a % of total detected for each sample) for occurrence of post-translational modifications as detected in the proteomics results for the three IAV samples. Only the PTMs that were detected >0.5% of the time were plotted.

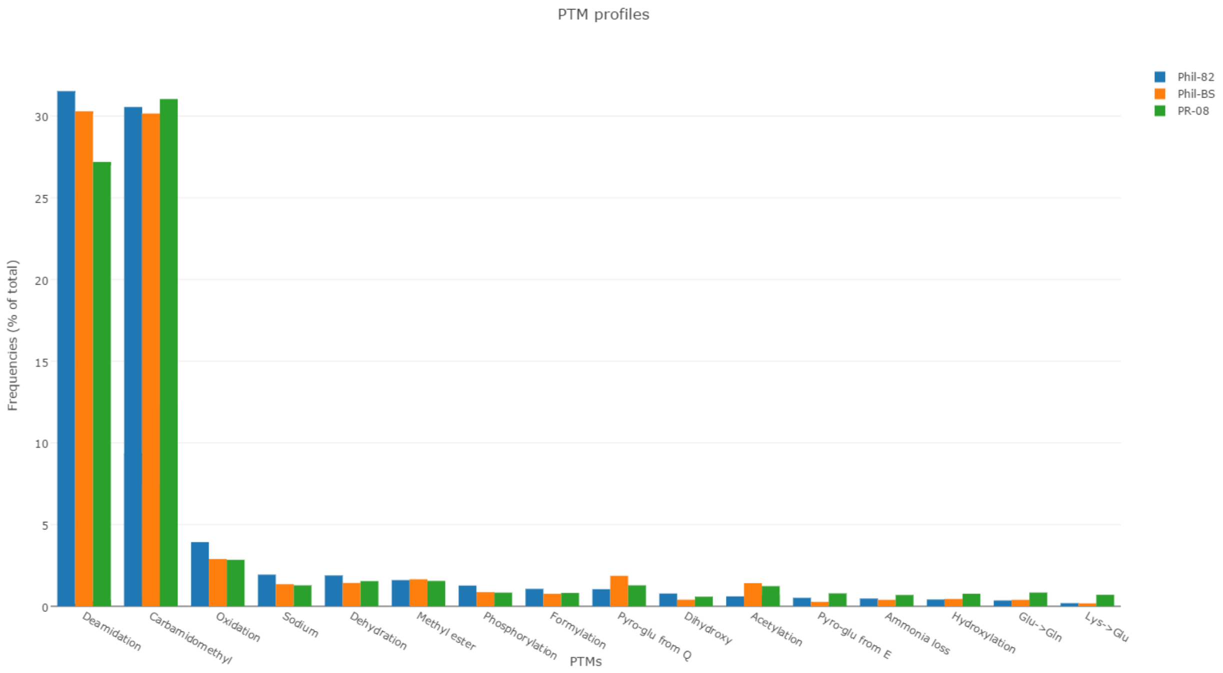

Supplemental Figure 3:

Site-specific glycoform distributions

Phil-82

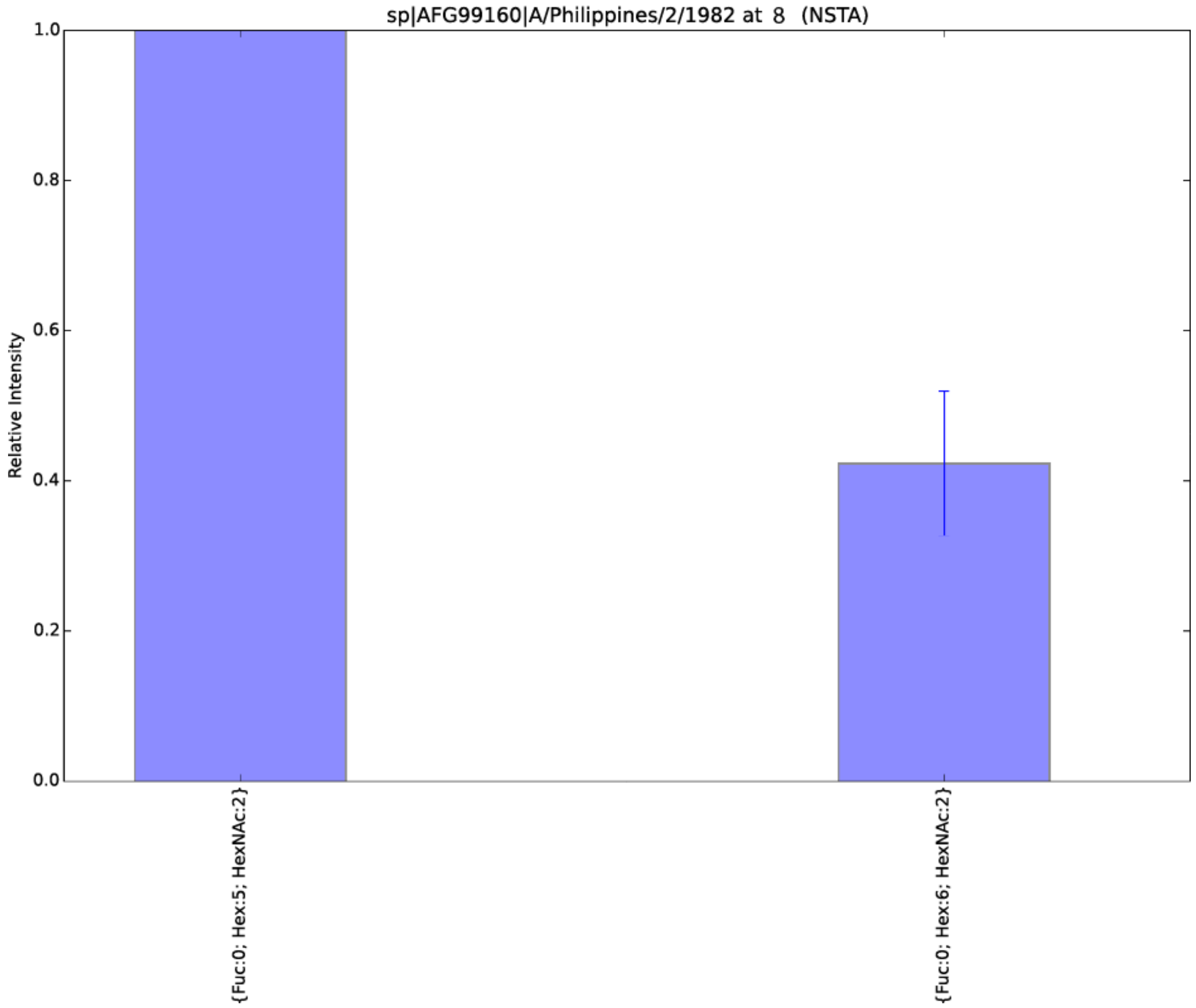

Supplemental Figure 3 (continued):

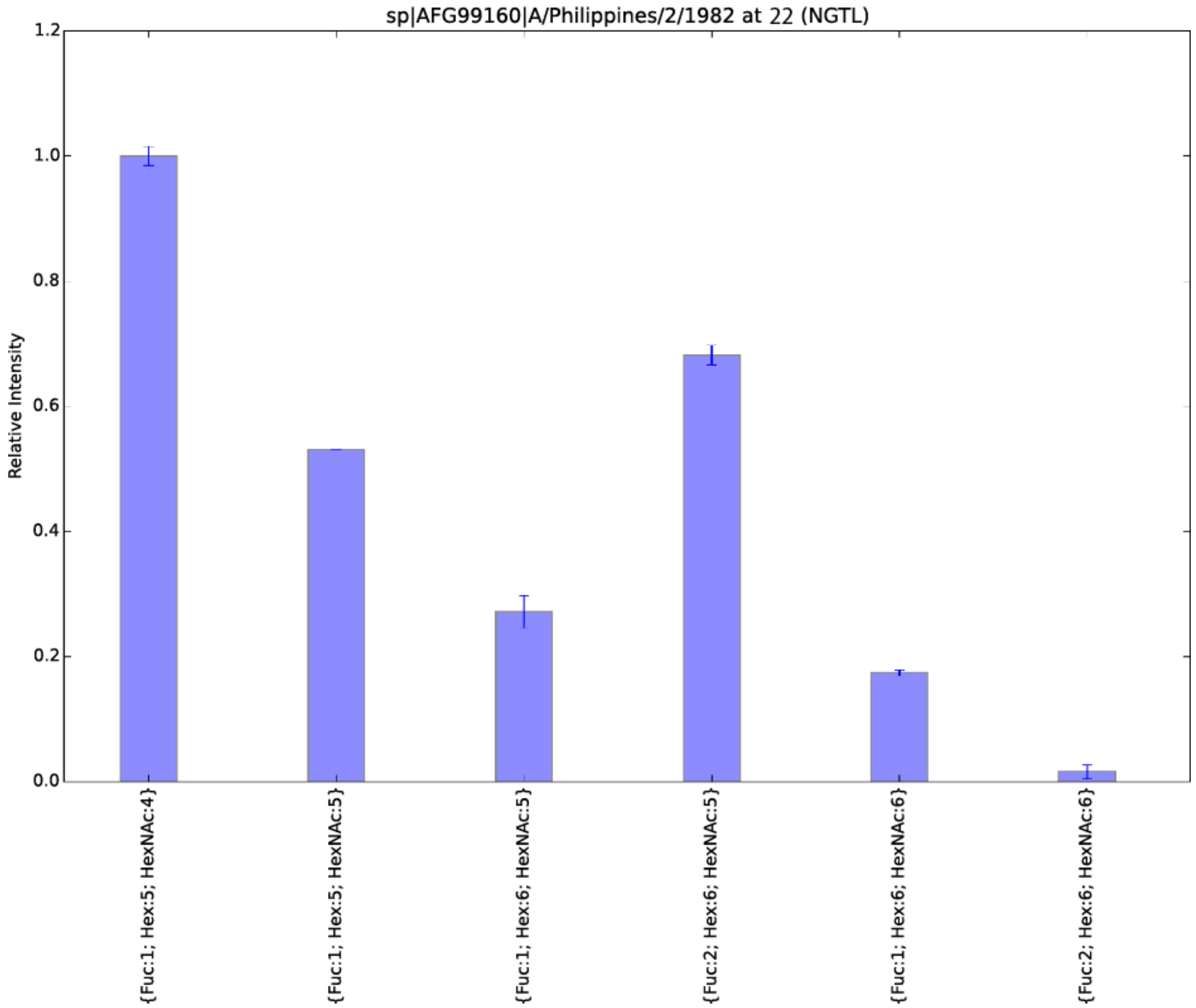

Supplemental Figure 3 (continued):

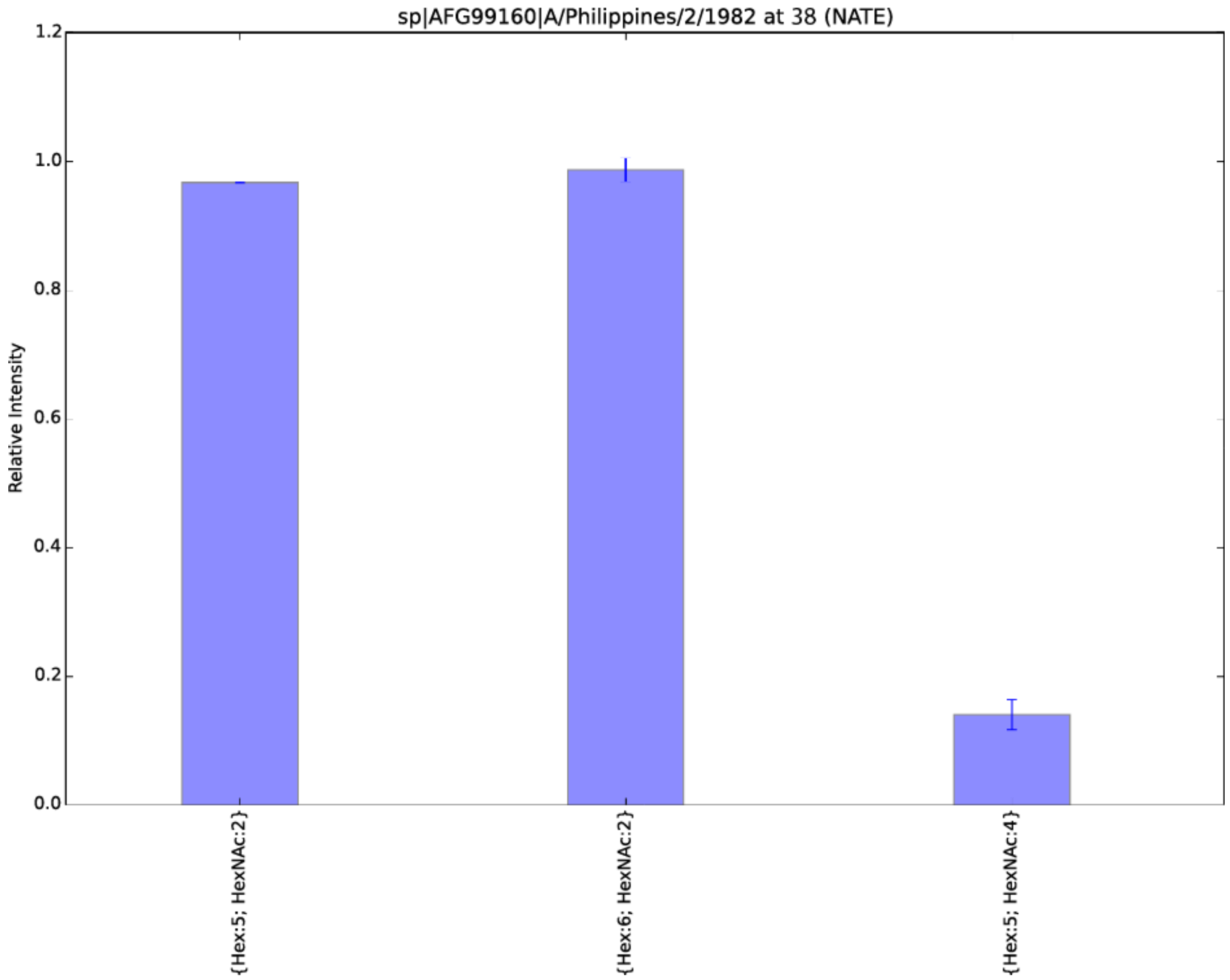

Supplemental Figure 3 (continued):

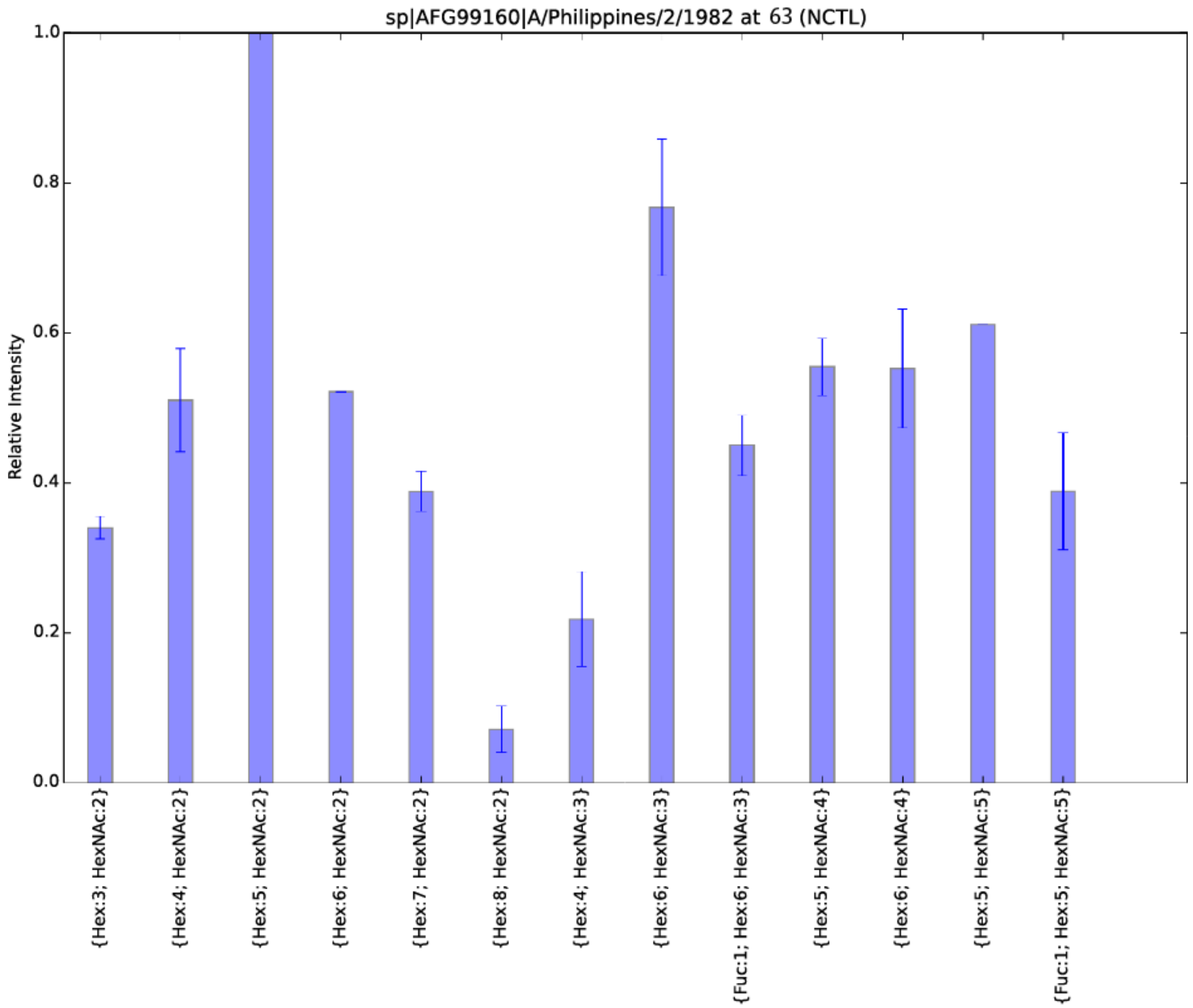

Supplemental Figure 3 (continued):

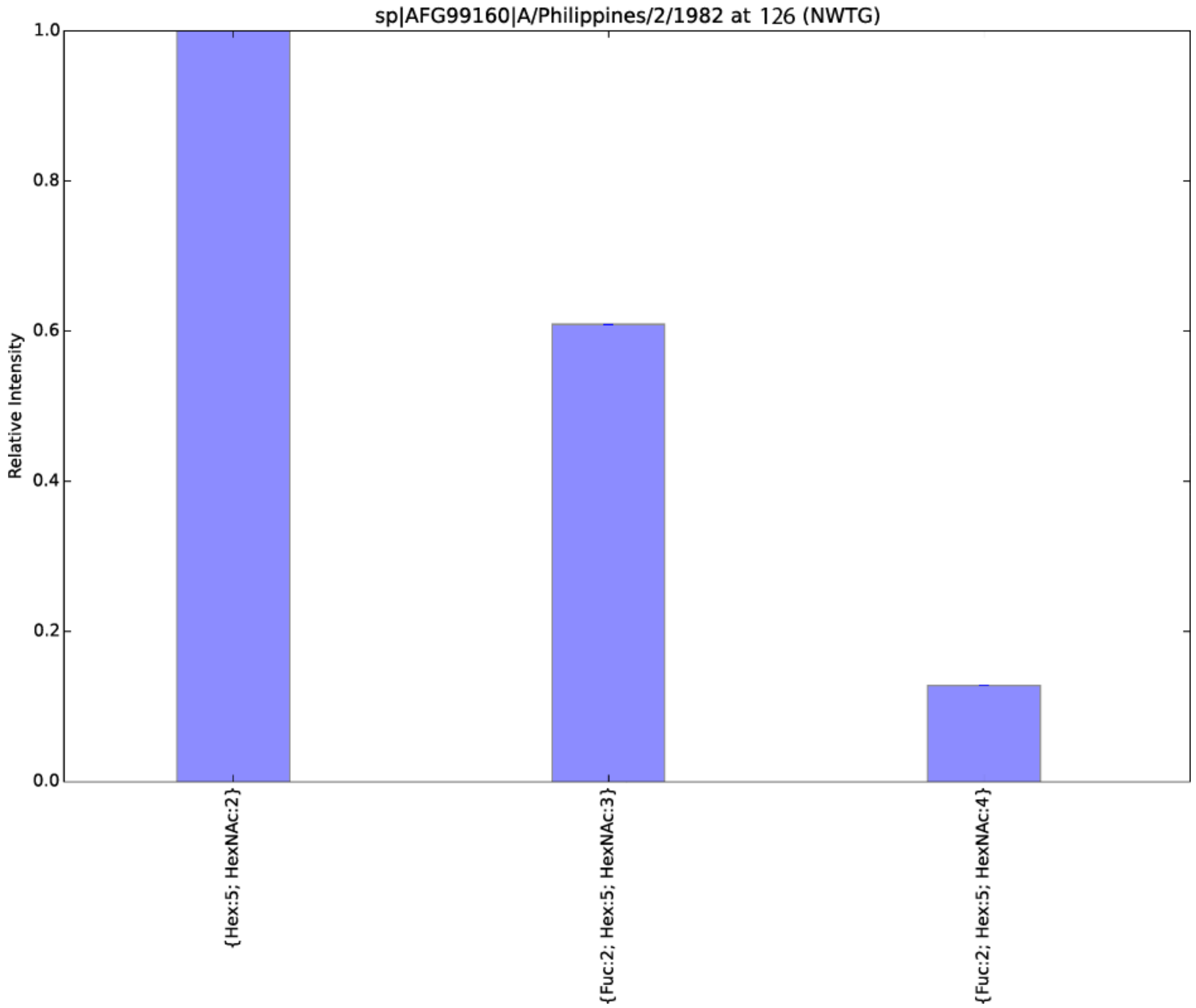

Supplemental Figure 3 (continued):

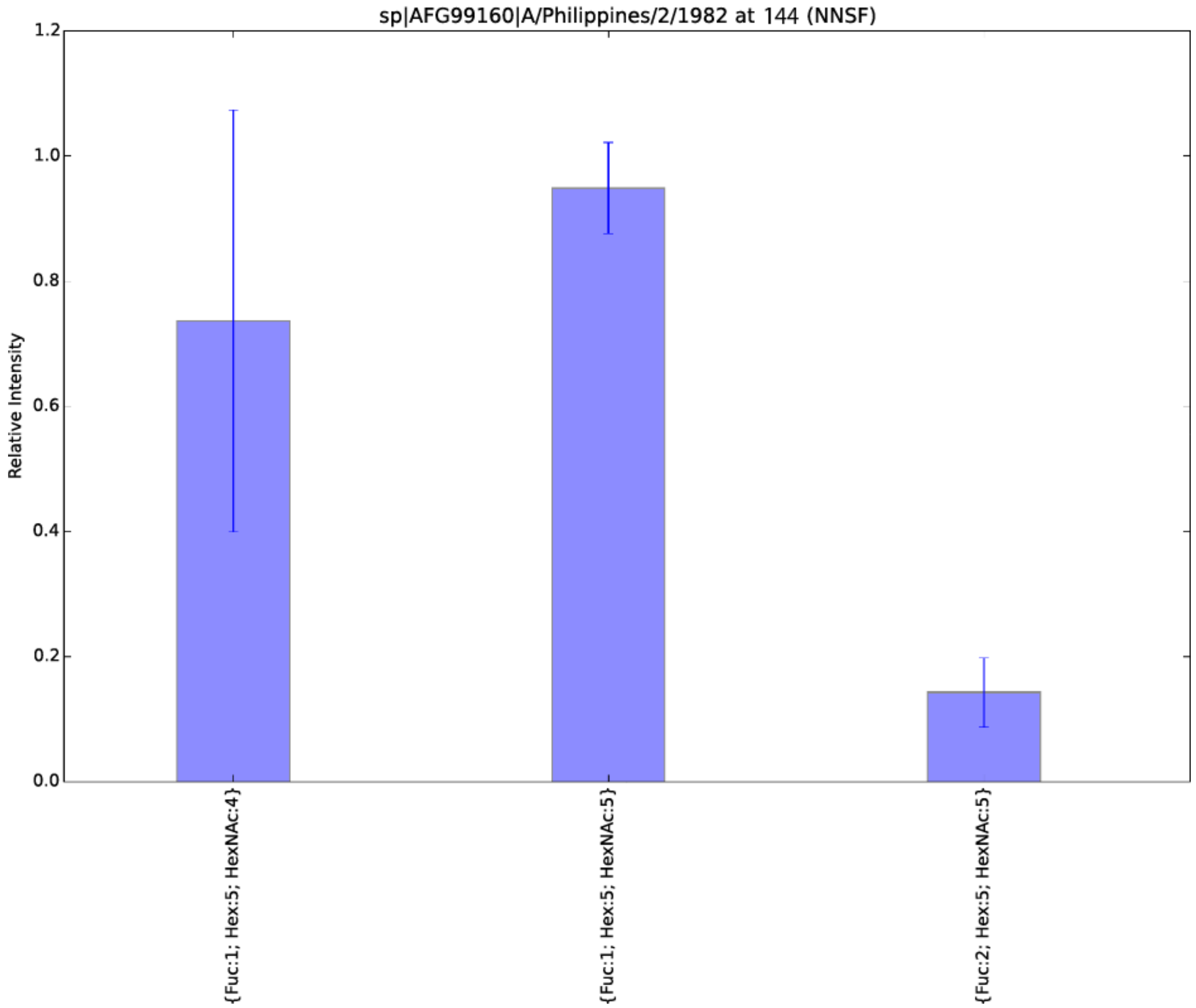

Supplemental Figure 3 (continued):

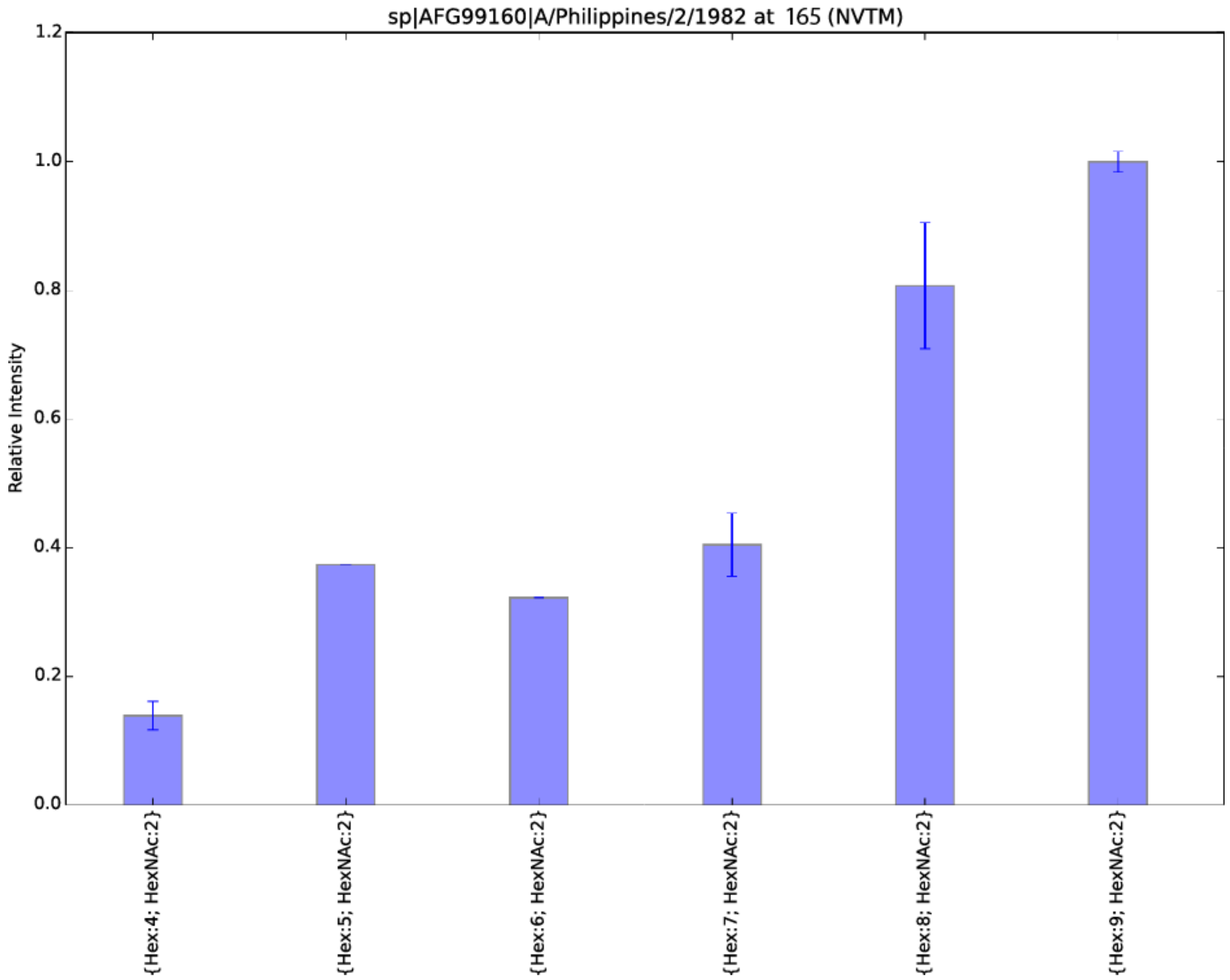

Supplemental Figure 3 (continued):

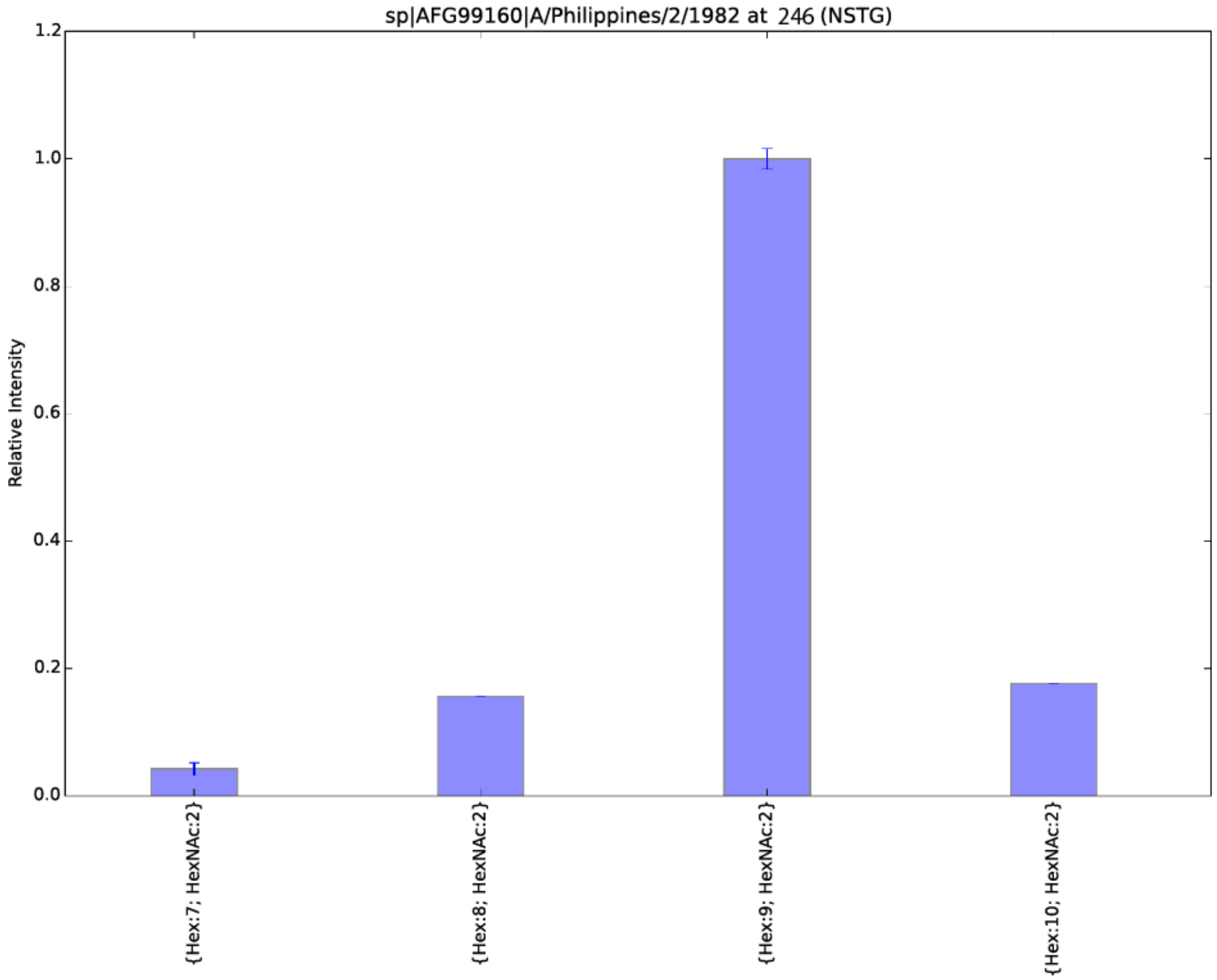

Supplemental Figure 3 (continued):

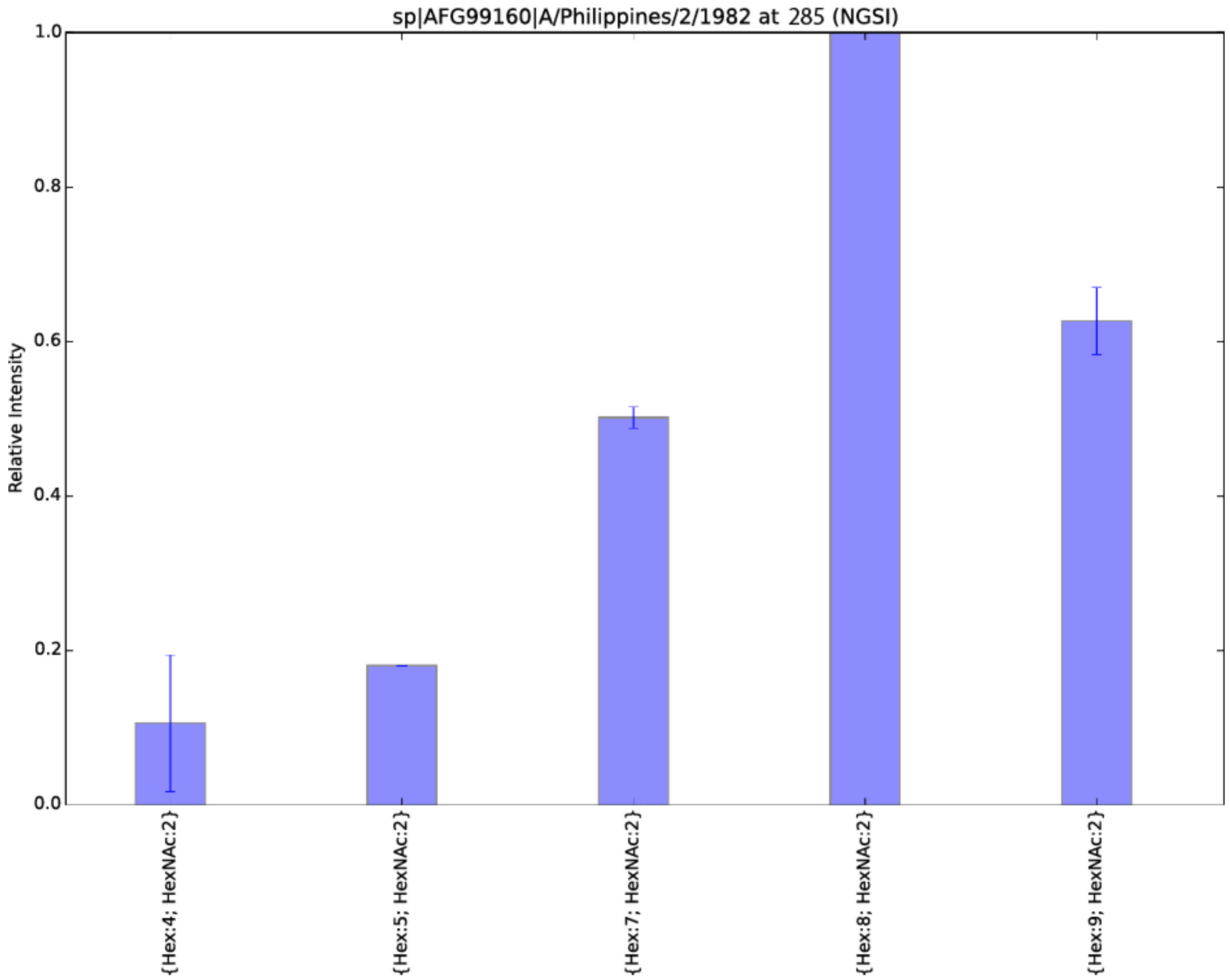

Supplemental Figure 3 (continued):

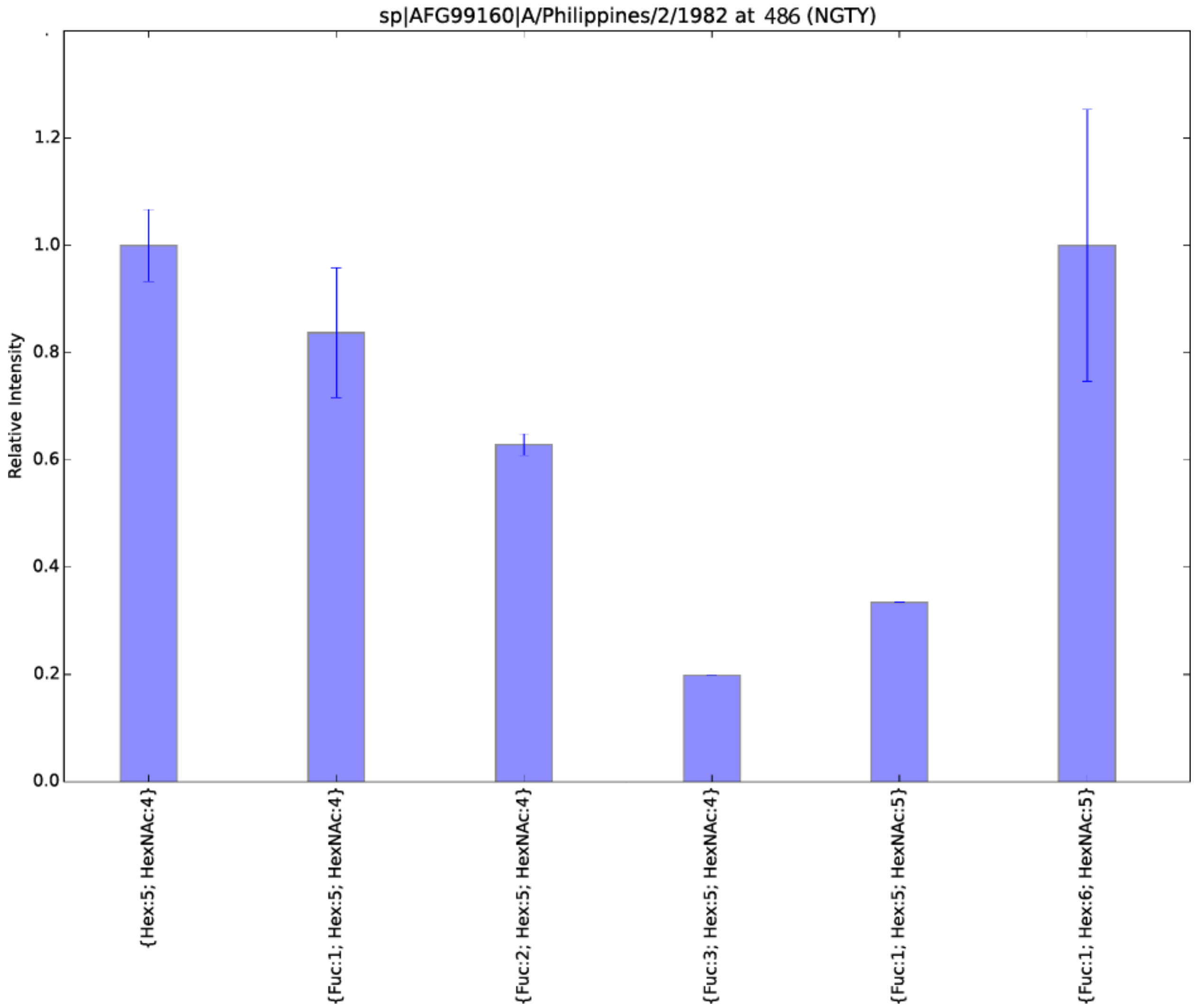

Supplemental Figure 3 (continued):

Phil-BS

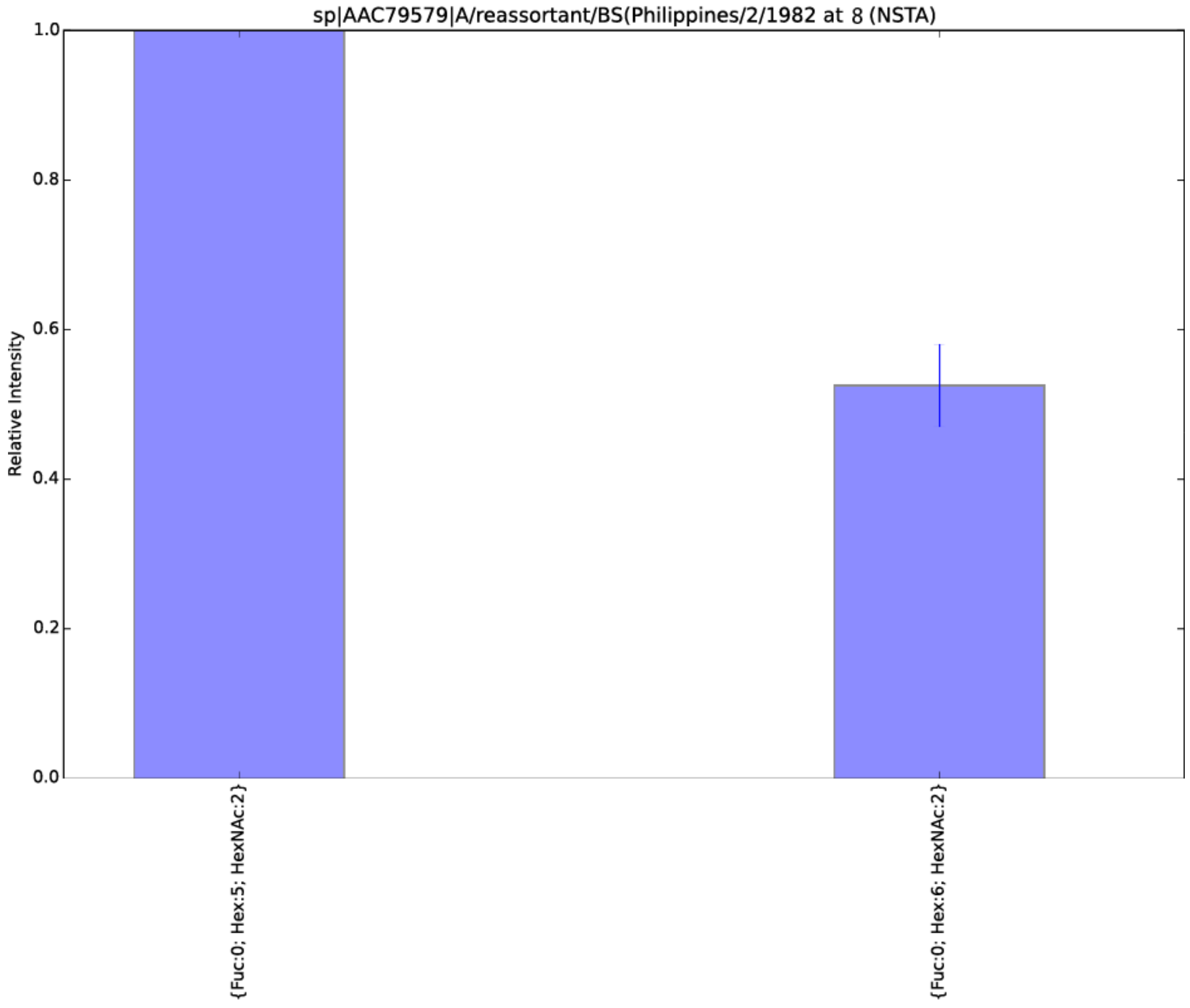

<u>Supplemental Figure 3 (continued):</u>

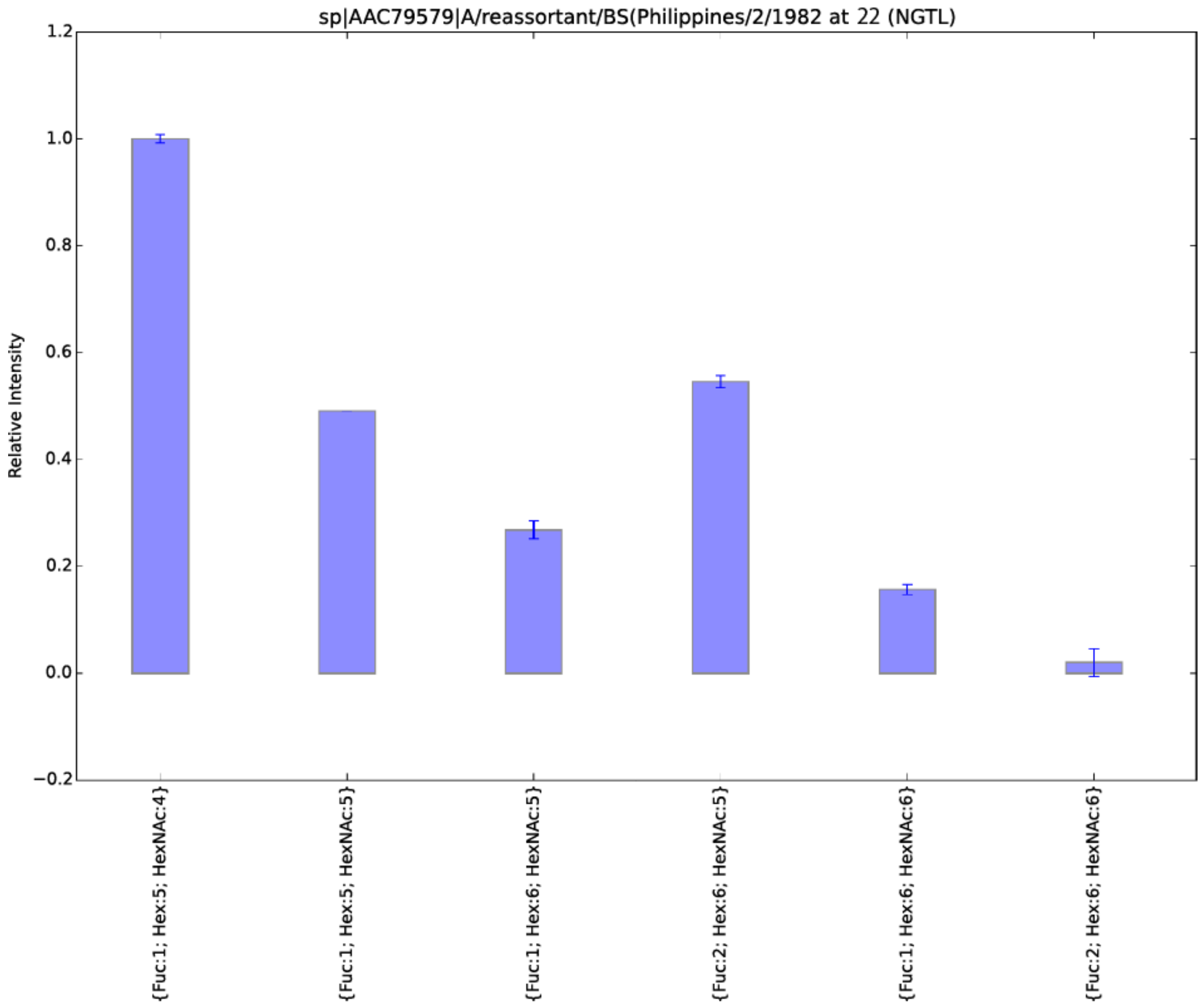

Supplemental Figure 3 (continued):

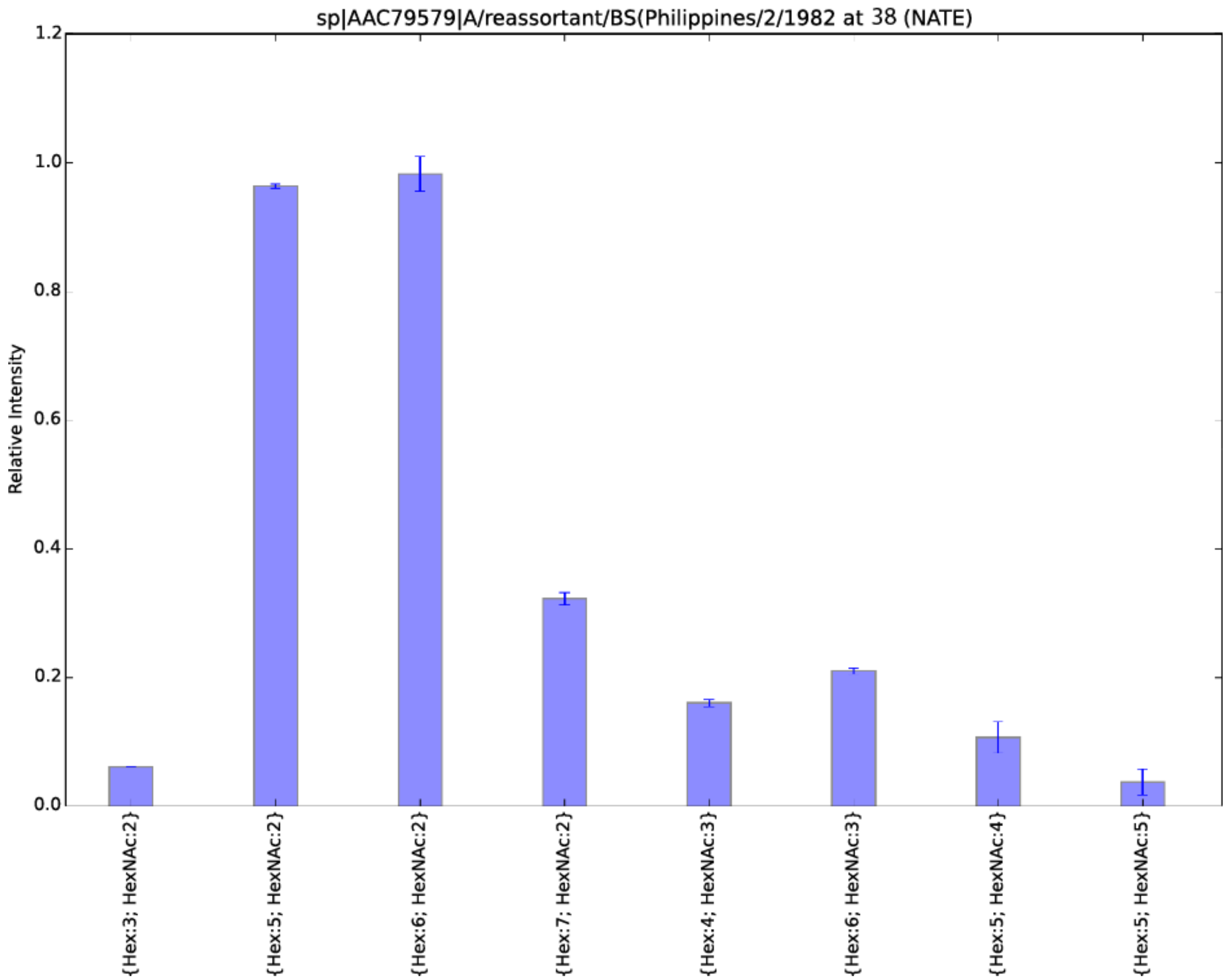

Supplemental Figure 3 (continued):

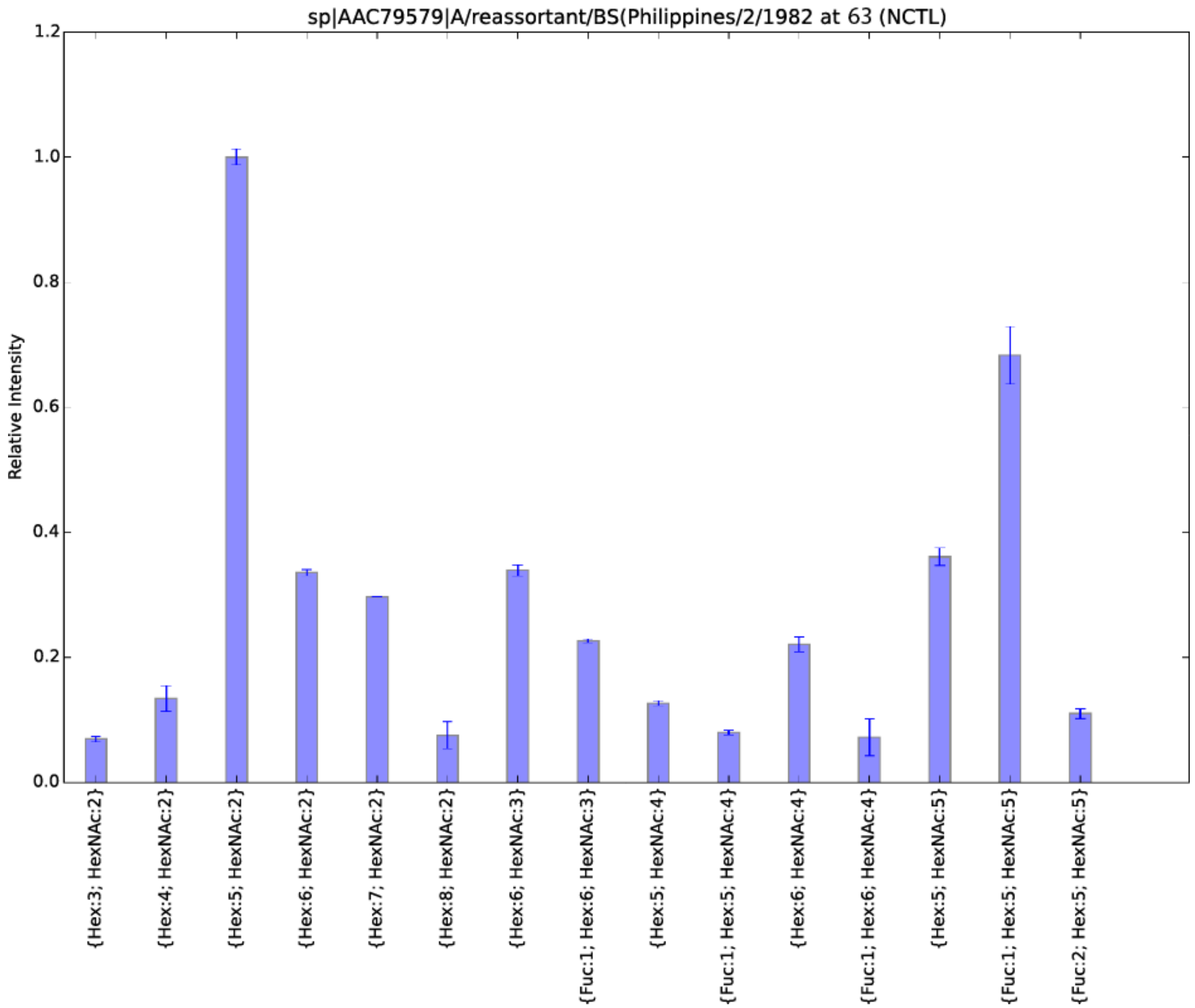

Supplemental Figure 3 (continued):

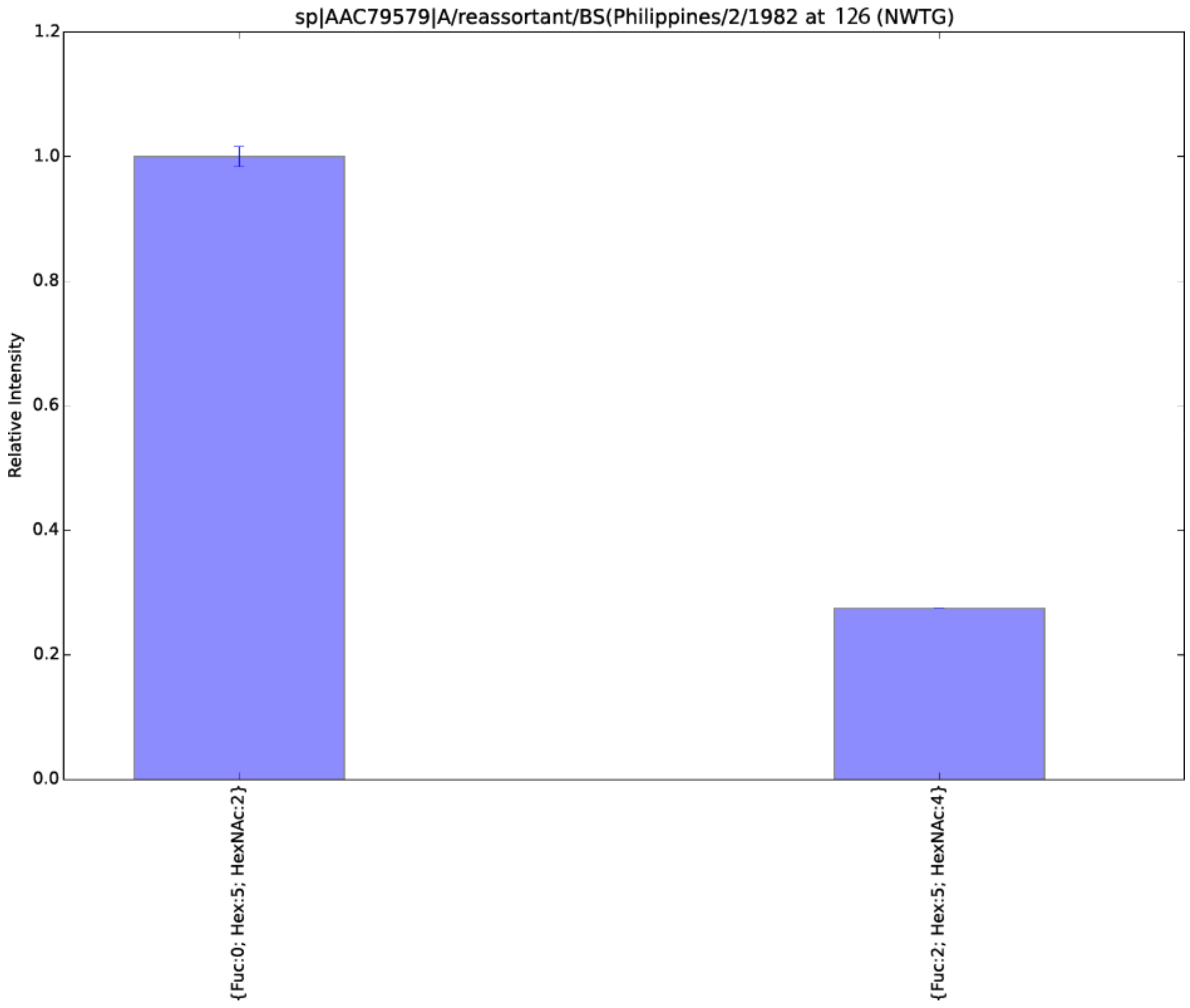

Supplemental Figure 3 (continued):

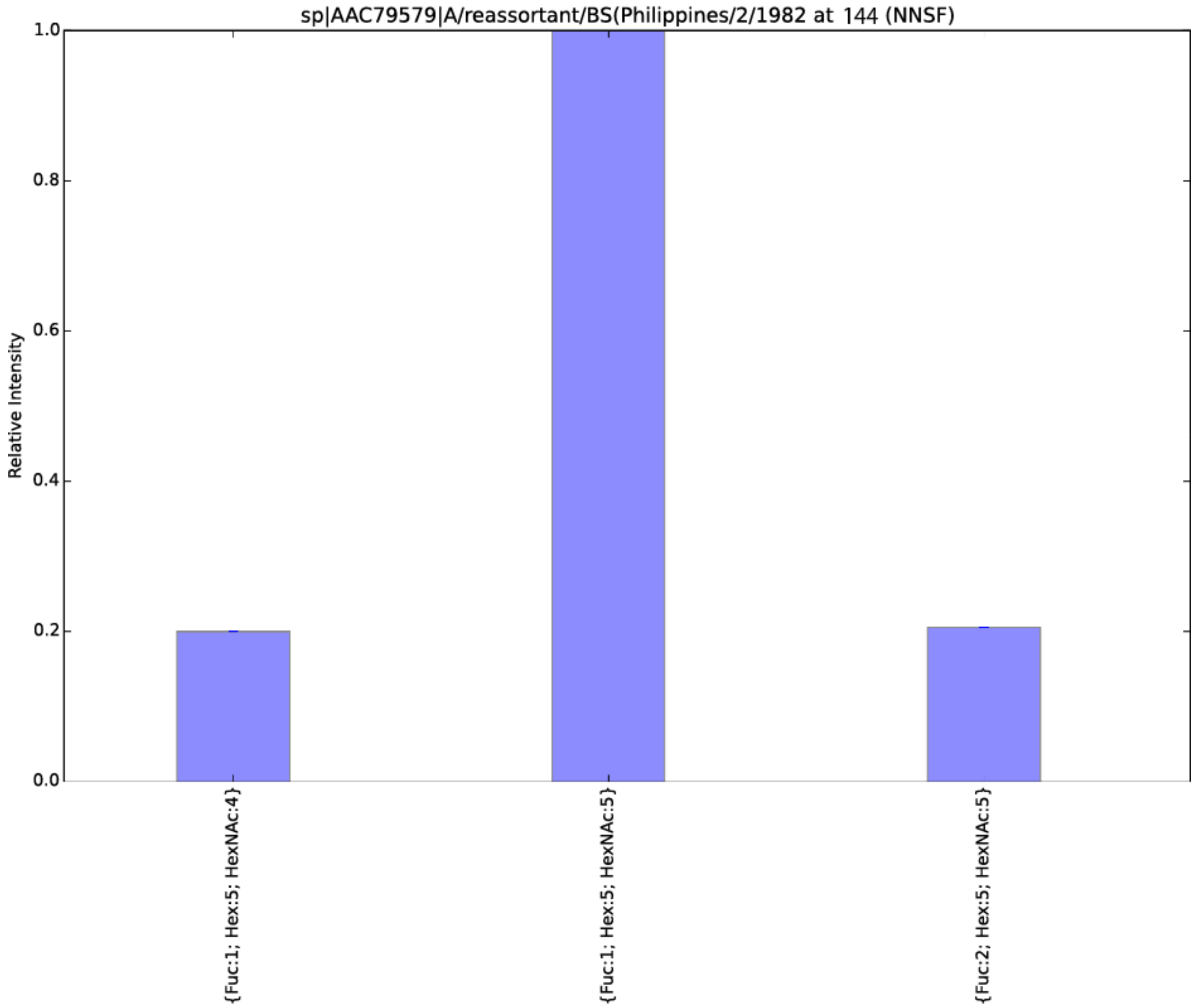

Supplemental Figure 3 (continued):

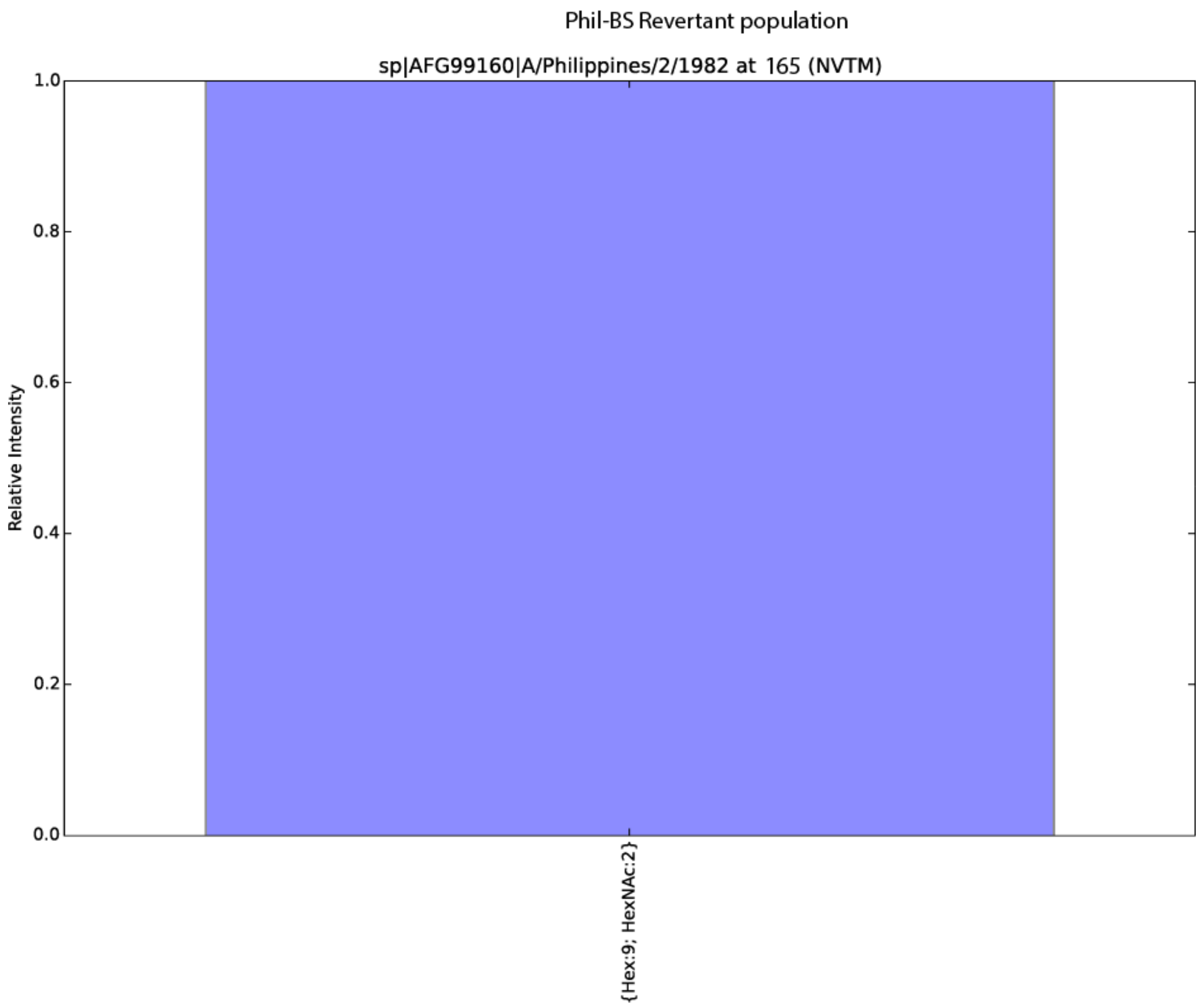

Supplemental Figure 3 (continued):

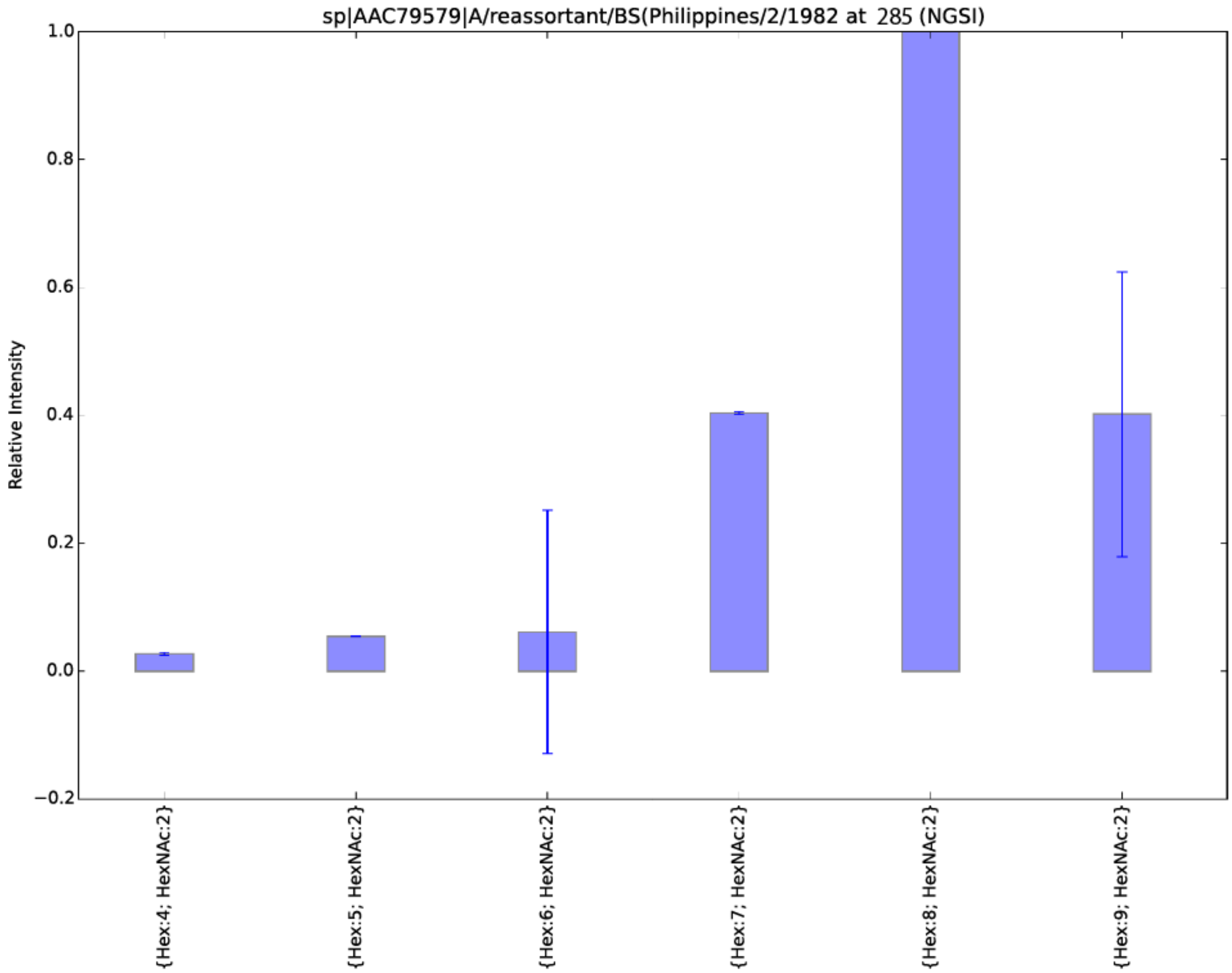

Supplemental Figure 3 (continued):

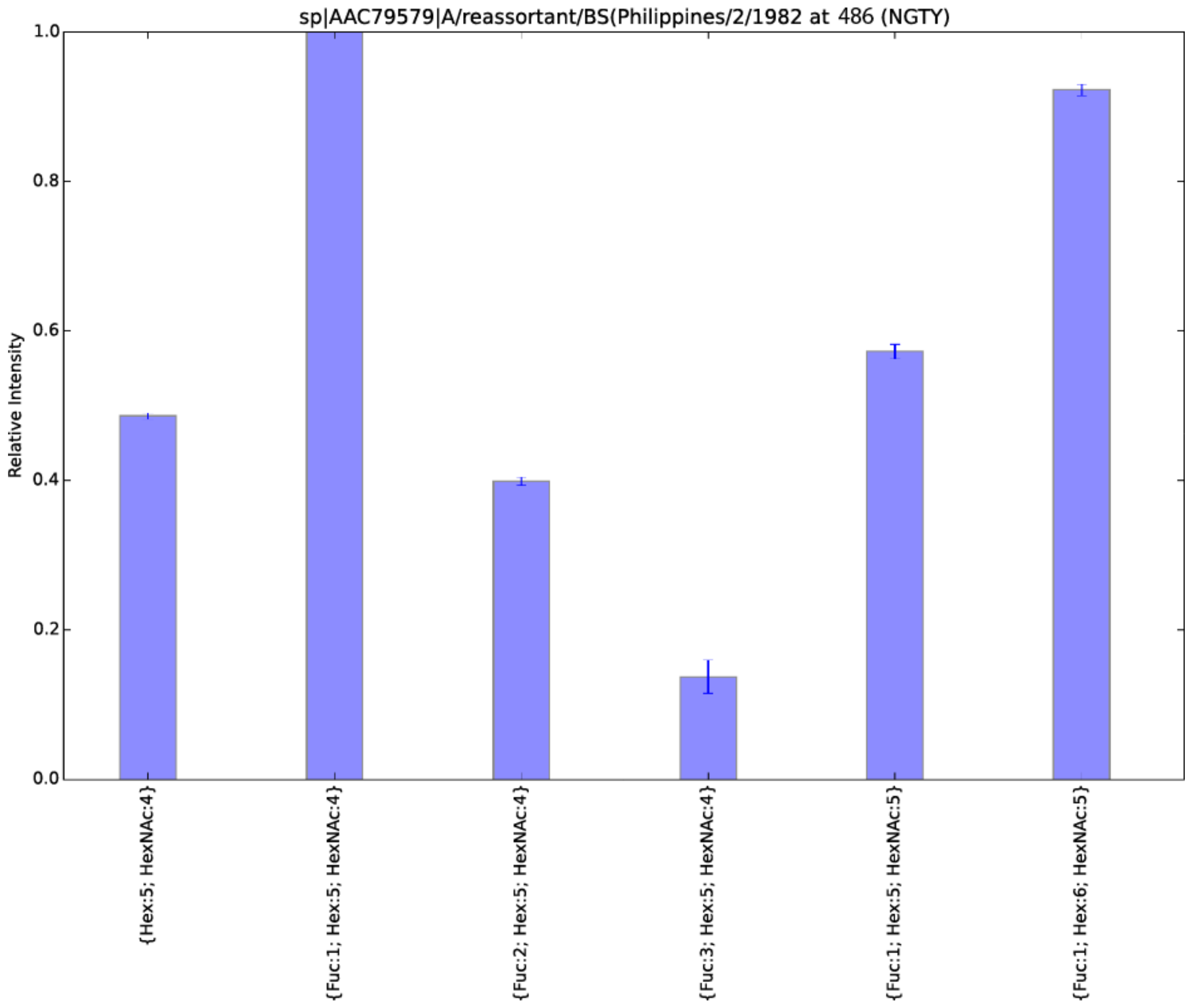

Supplemental Figure 3 (continued):

PR-08

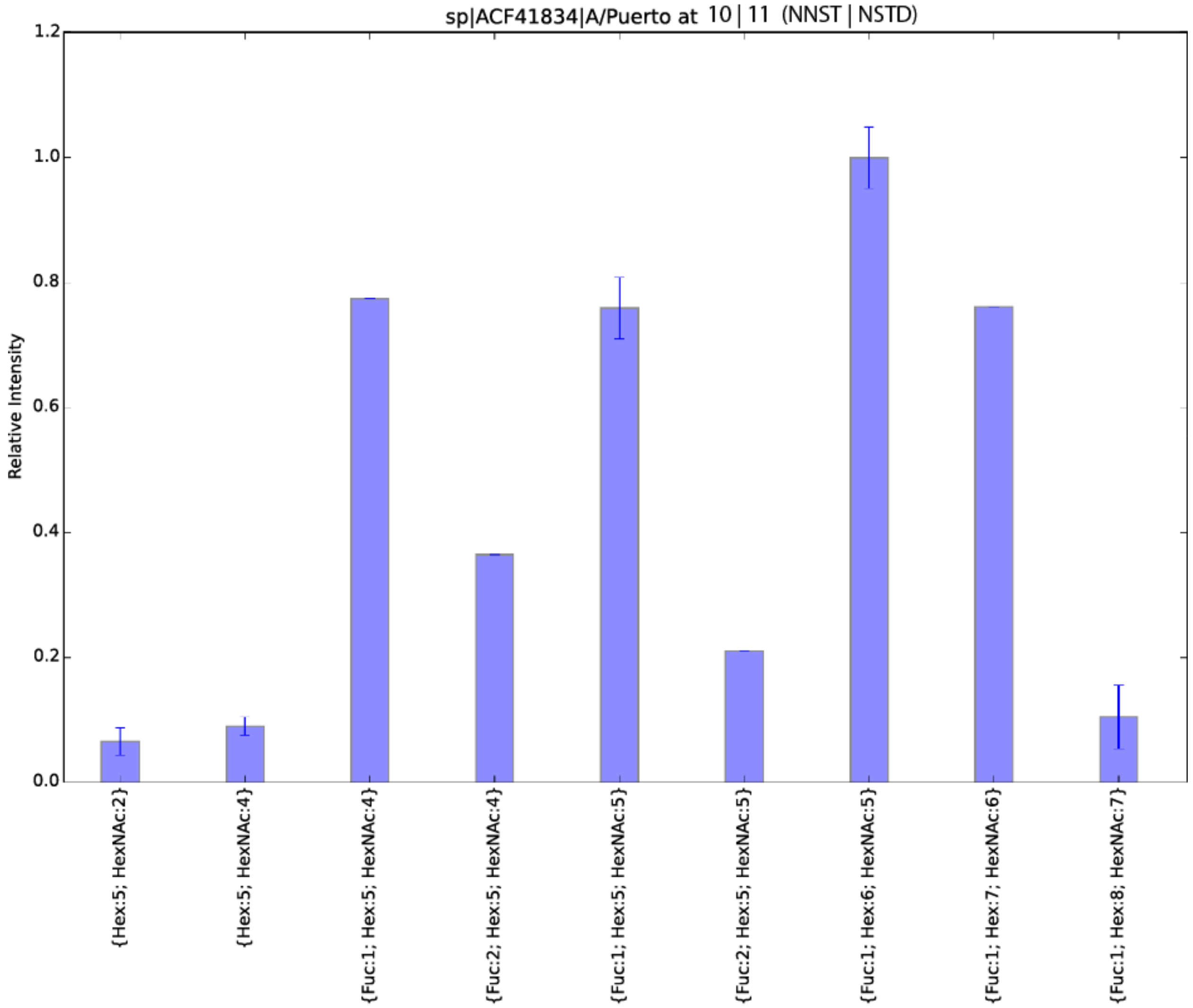

Supplemental Figure 3 (continued):

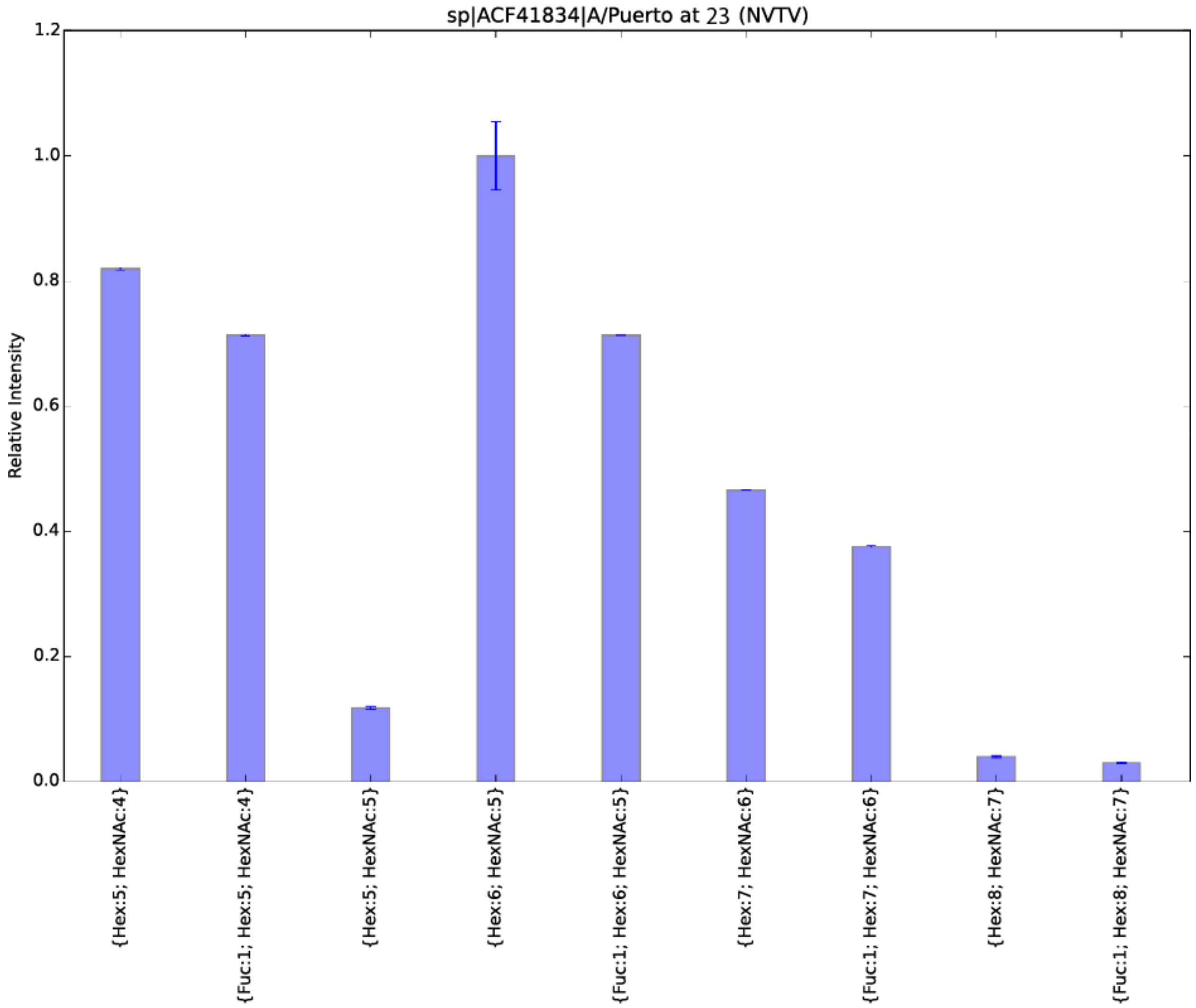

Supplemental Figure 3 (continued):

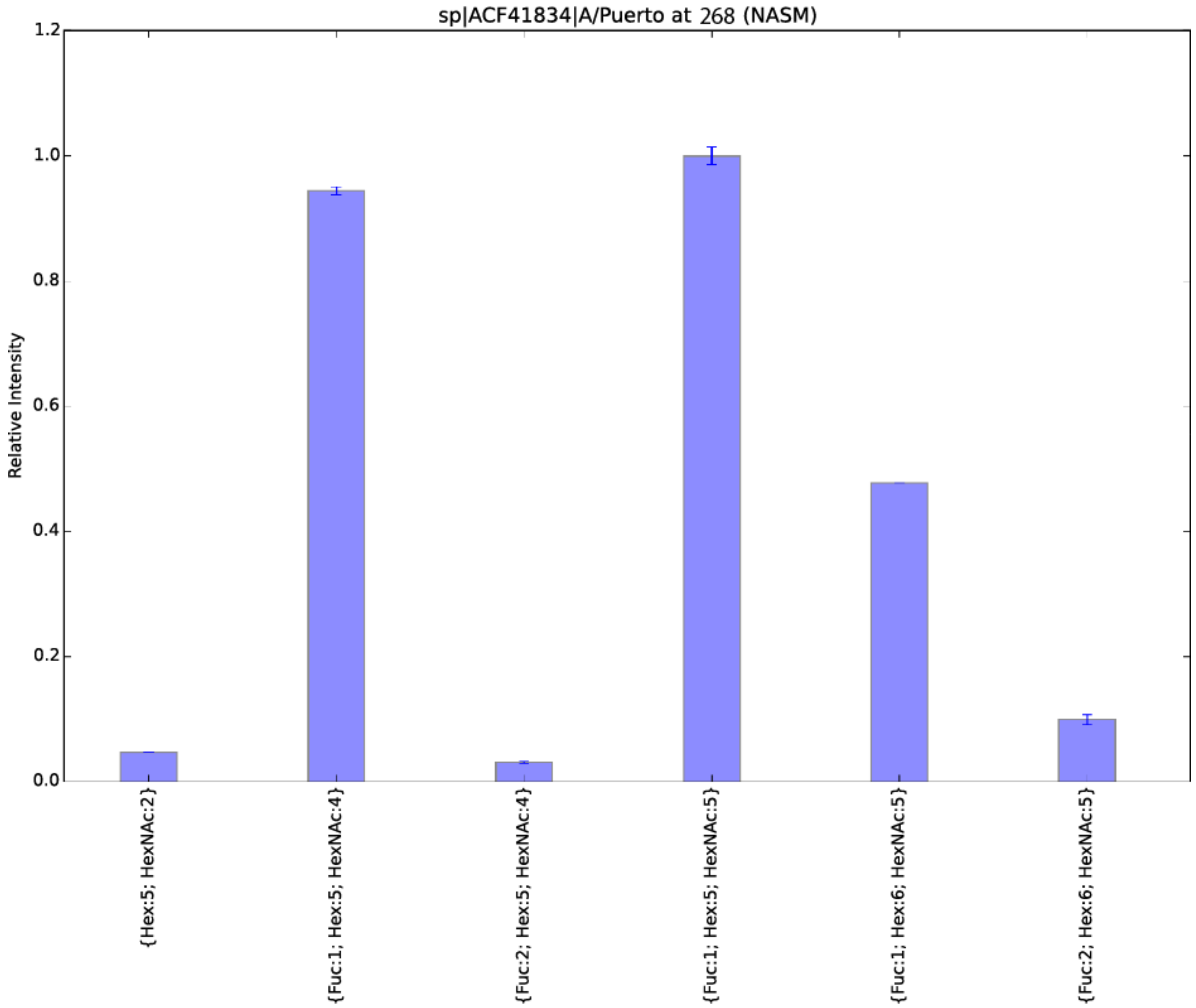

Supplemental Figure 3 (continued):

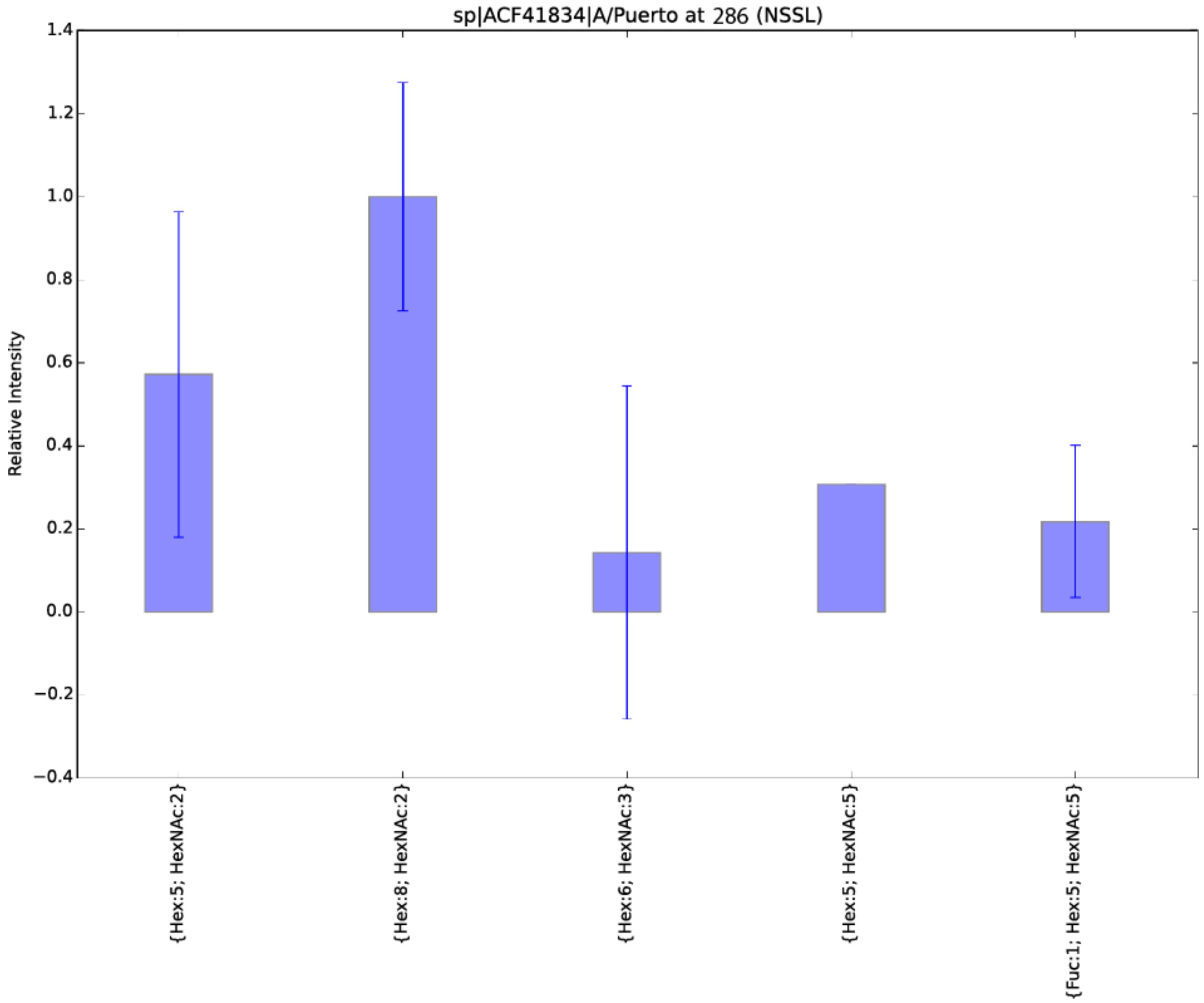

Supplemental Figure 3 (continued):

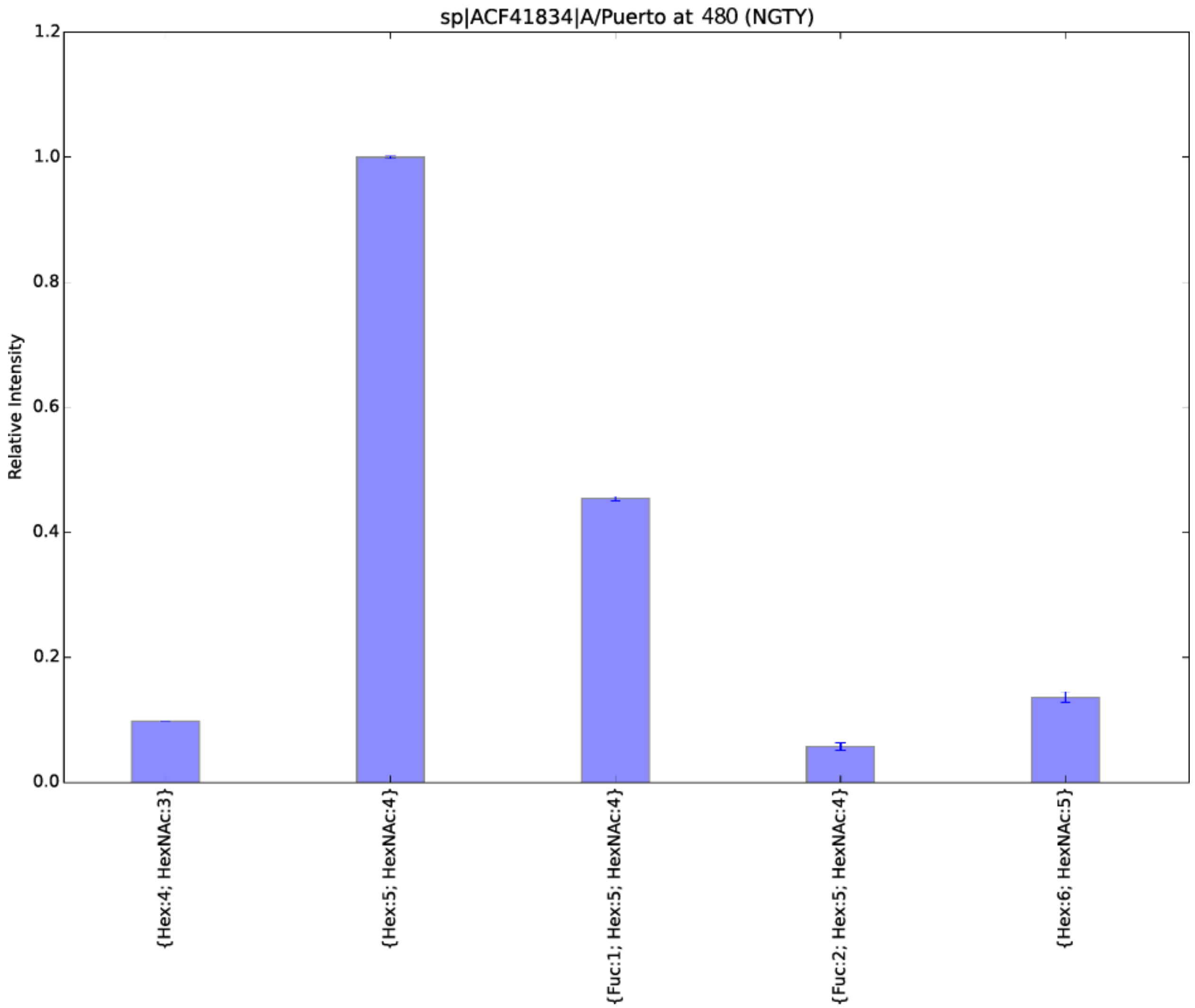

__Supplemental Figure 4: Site specific glycoforms:__

The following figures show site-specific glycoform distributions for the three virus strains studied, aligned to the HA monomers for respective virions. Homology model for Phil-82 and Phil-BS hemagglutinins was generated as described in the main text. PR-08 model was generated using the 1934 Human H1 Hemagglutinin structure from PDB ID: 1RU7. (NA: Not available)

Supplemental figure 4 A: Phil-82

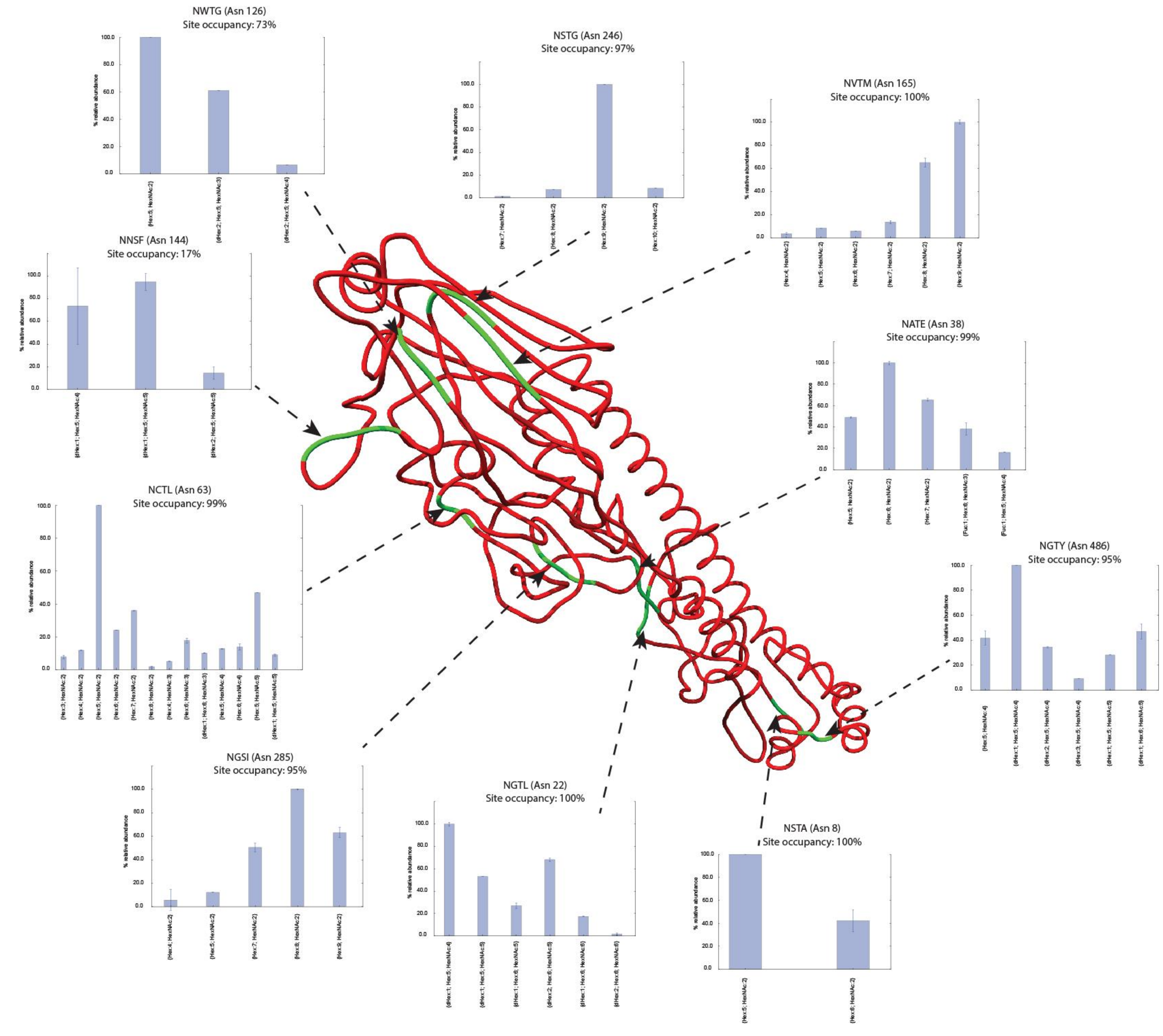

Supplemental figure 4B: Phil-BS

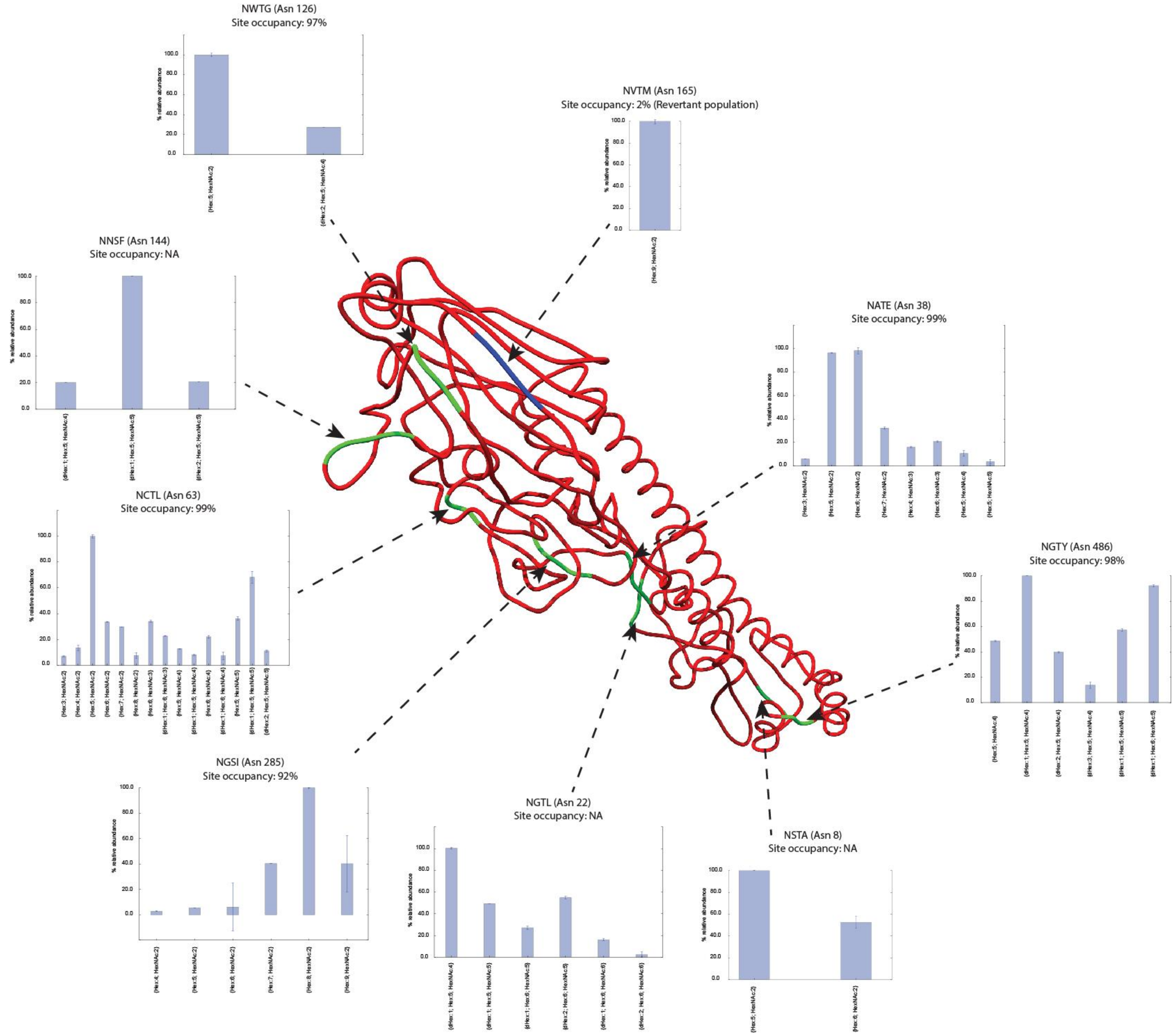

Supplemental figure 4C: PR-08

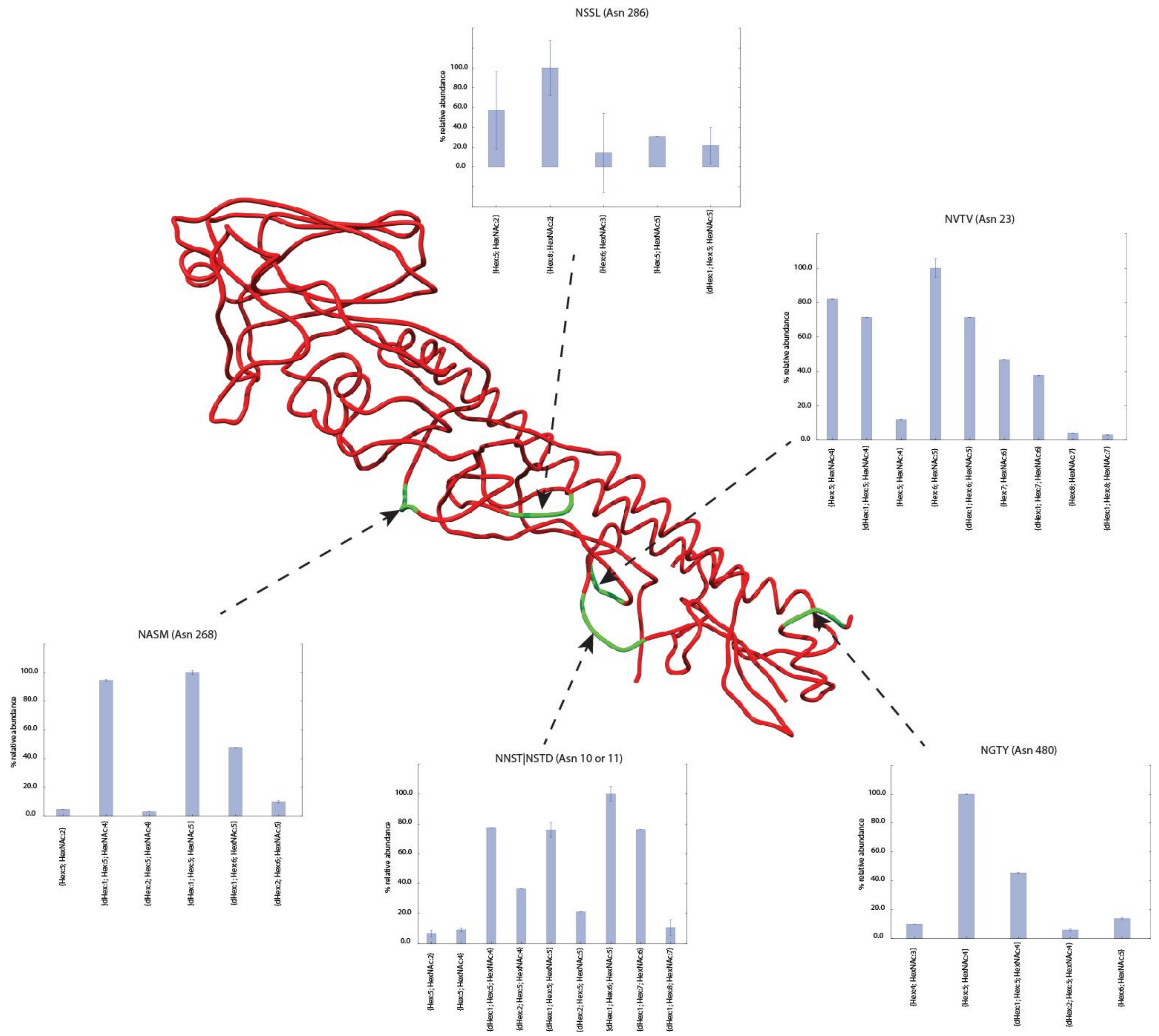

Supplemental Figure 5:

The following figures compare glycomics and glycopeptidomics data for overall glycoform distributions. Glycans are categorized by number of HexNAc units in matched compositions. Glycoform abundances from glycopeptides were generated by summing all abundances for glycopeptide matches confirmed by tandem MS, with the specified number of HexNAc units. Glycoform abundances from glycomics were plotted using MS1 profiling data.

Supplemental figure 5A: Phil-82

Phil-82 glycan distribution from Glycoproteomics

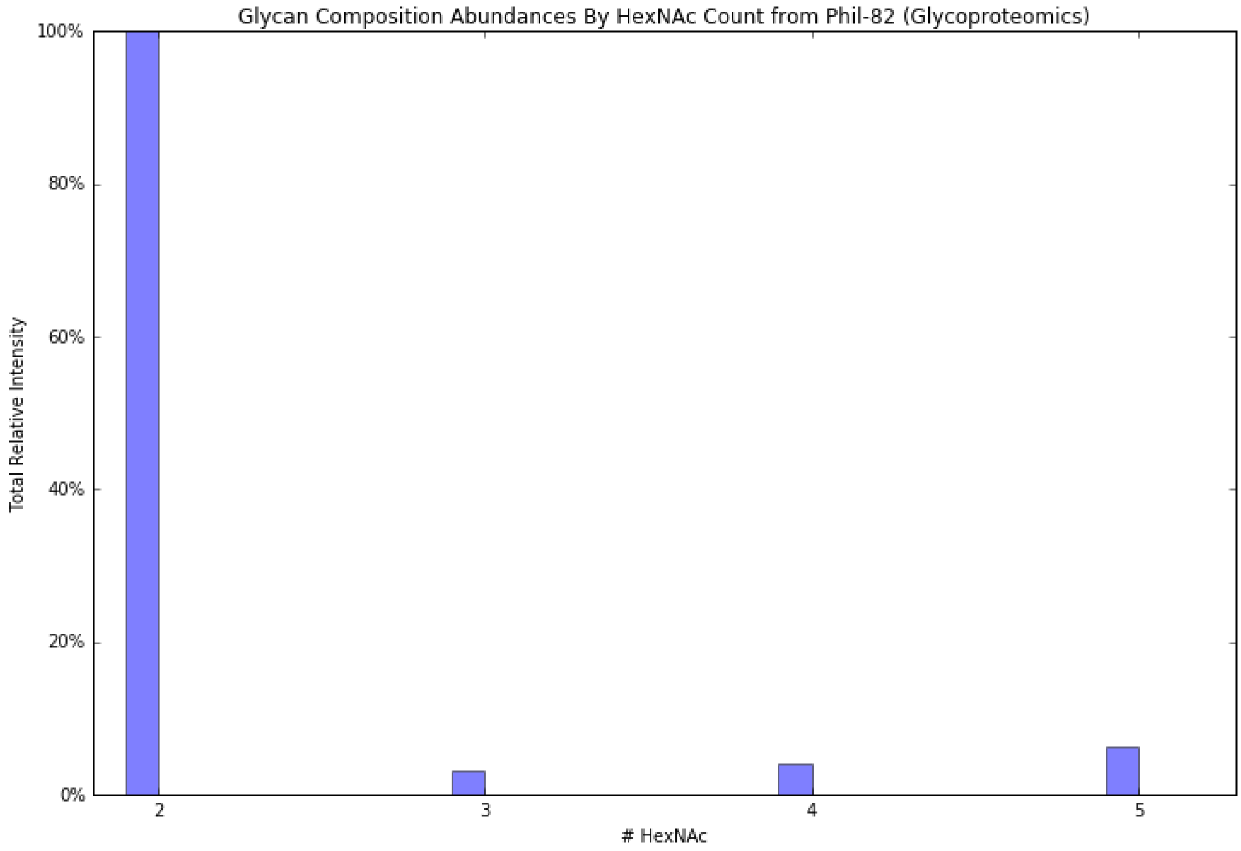

Phil-82 glycan distribution from Glycomics

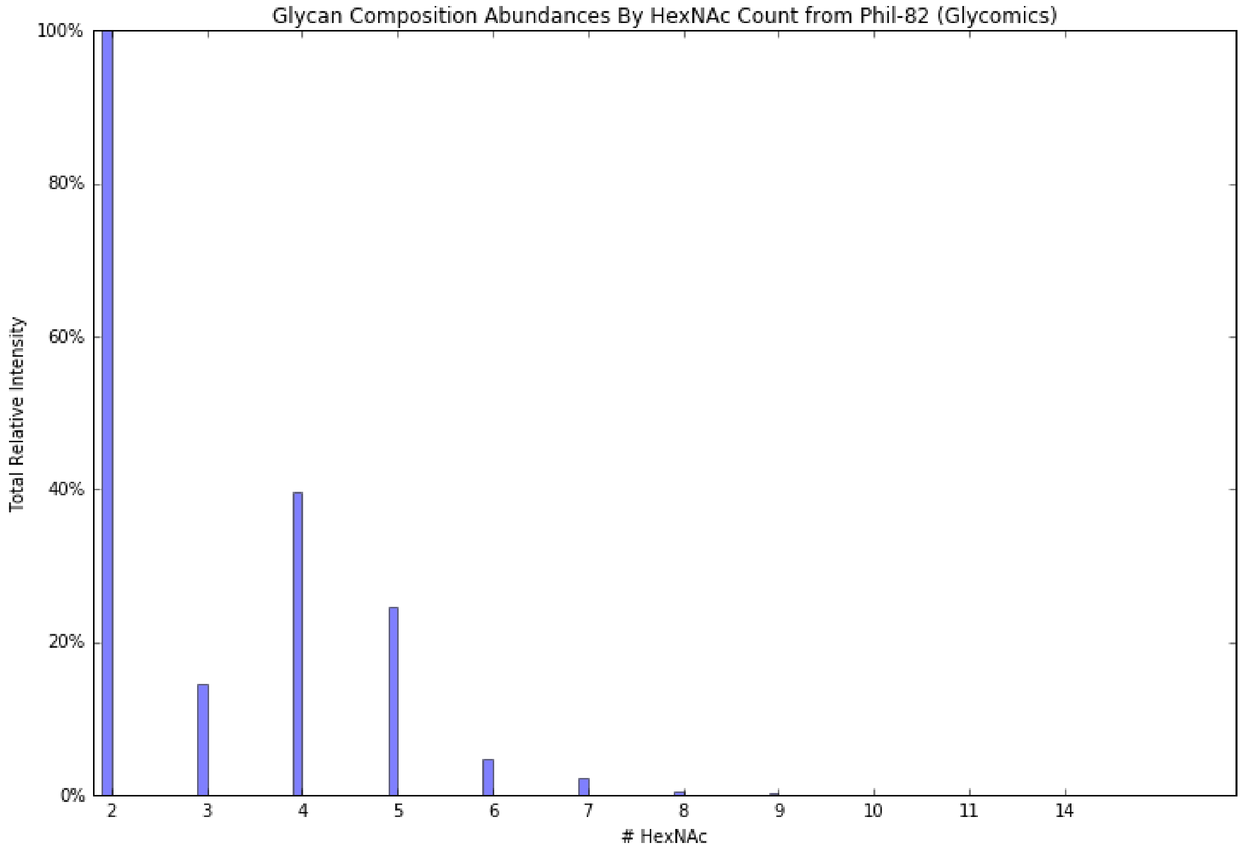

Supplemental figure 5B: Phil-BS

<u>Phil-BS glycan distribution from Glycoproteomics</u>

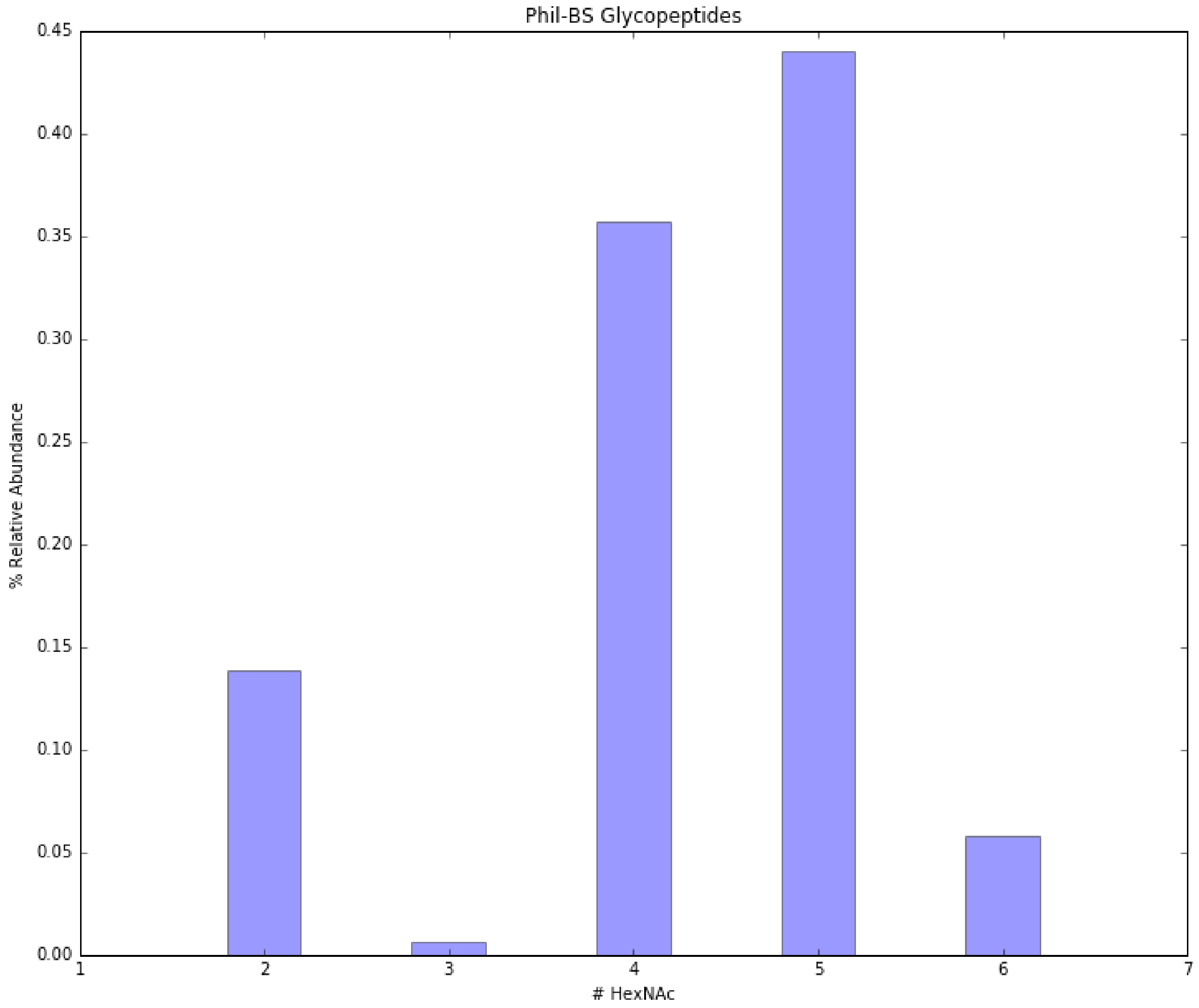

Phil-BS glycan distribution from Glycomics

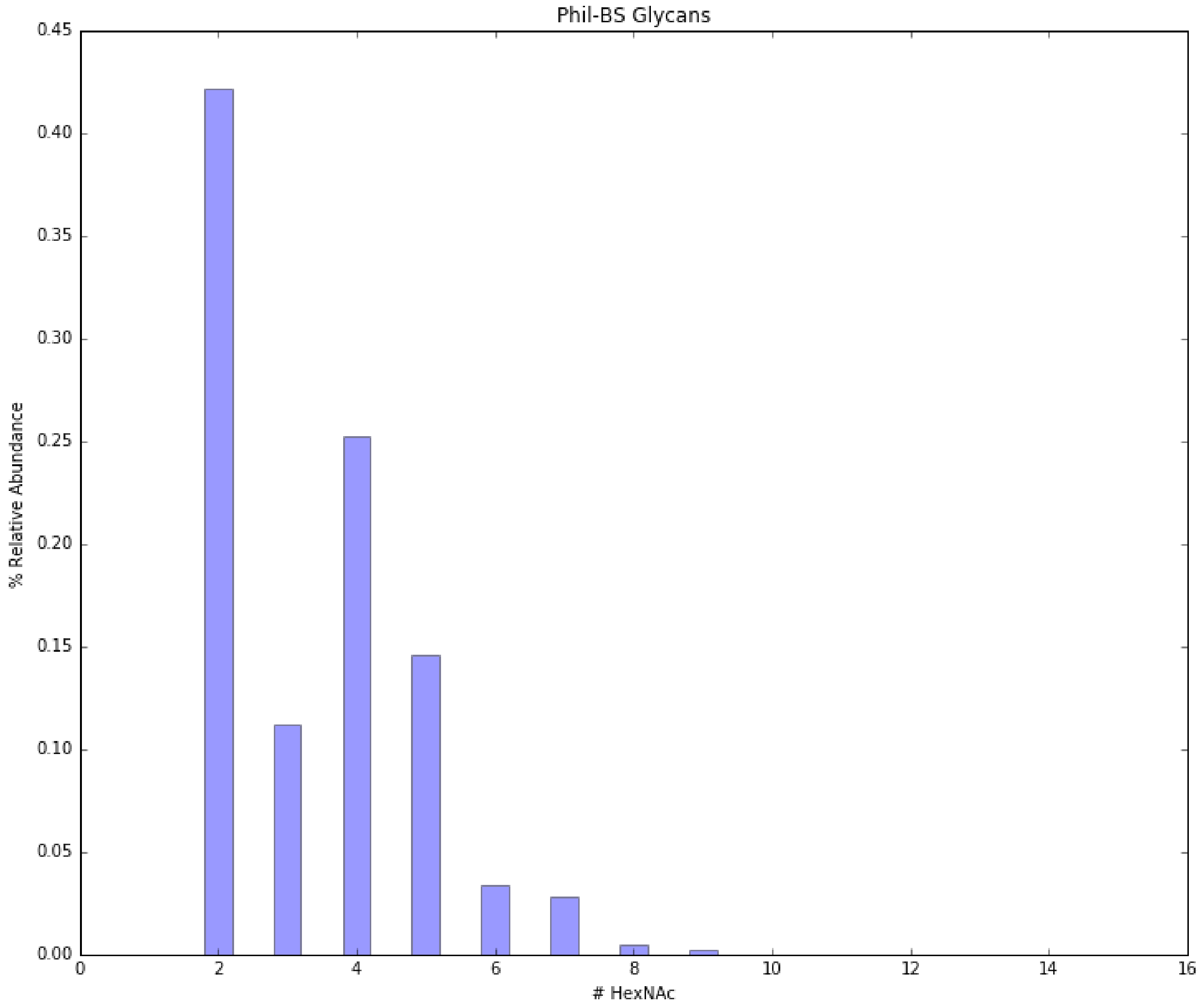

Supplemental figure 5C: PR-08

PR-08 glycan distribution from Glycoproteomics

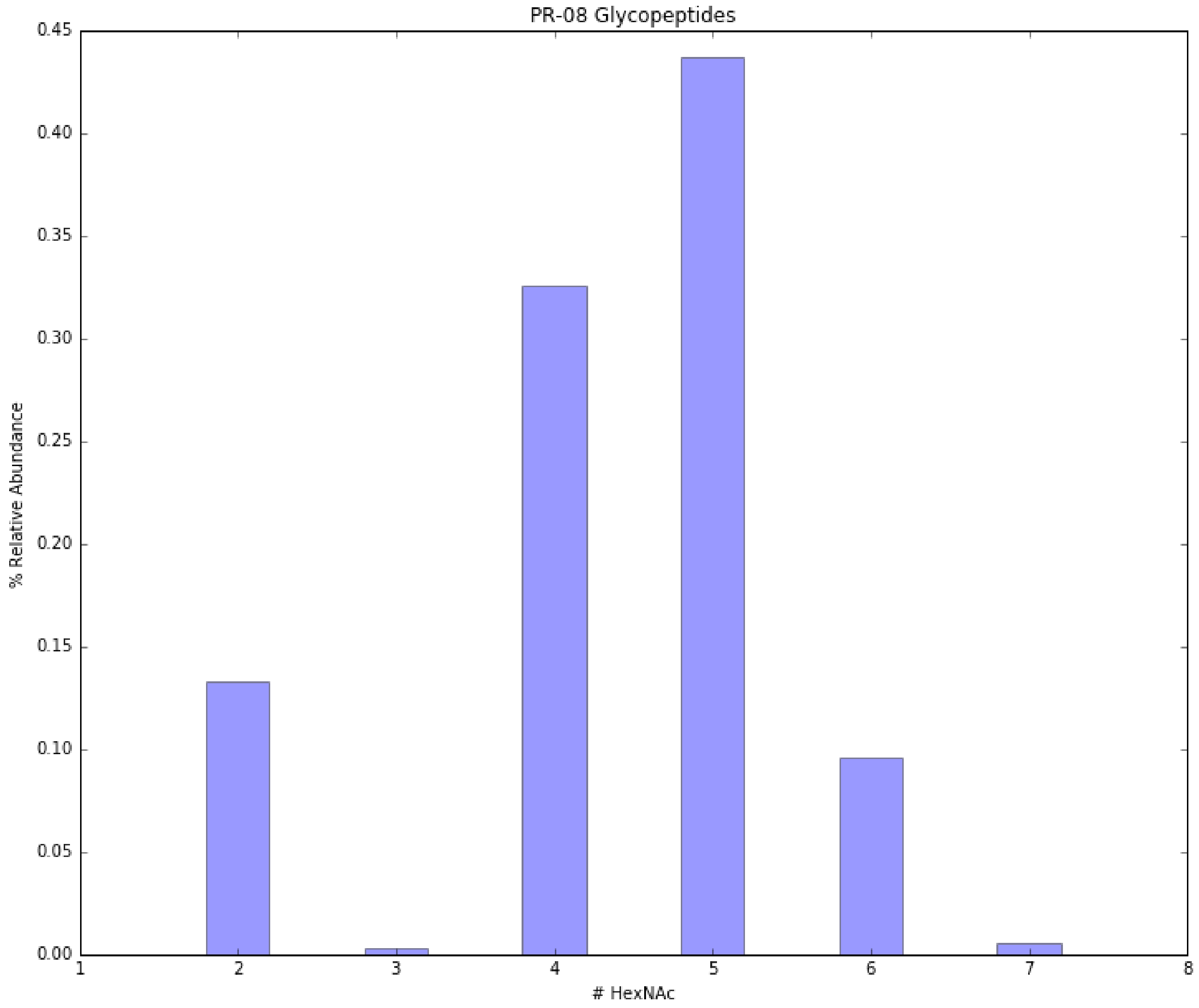

PR-08 glycan distribution from Glycomics

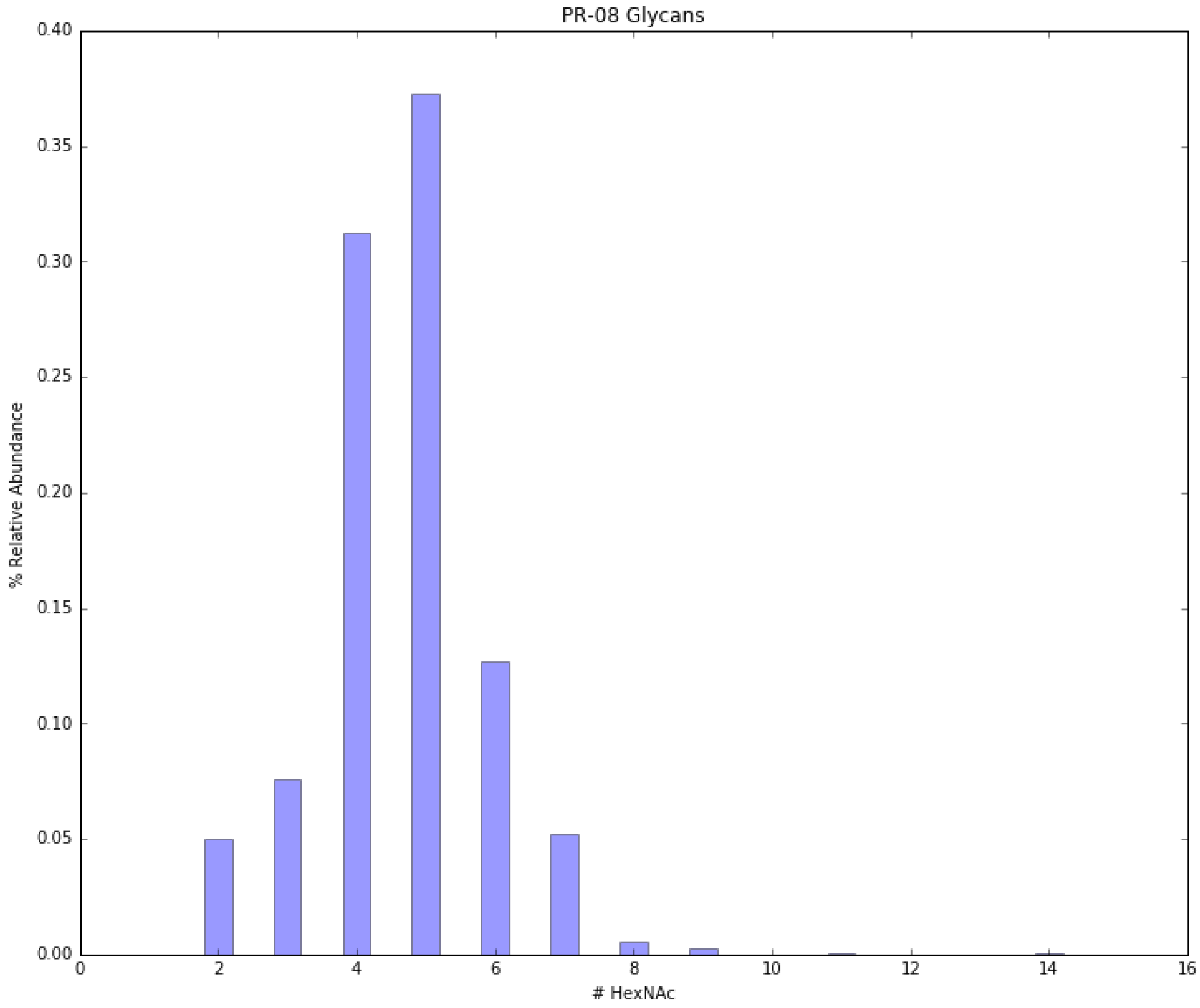

References cited:

1. Maxwell, E., Tan, Y., Tan, Y., Hu, H., Benson, G., Aizikov, K., Conley, S., Staples, G. O., Slysz, G. W., Smith, R. D., and Zaia, J. (2012) GlycReSoft: A Software Package for Automated Recognition of Glycans from LC/MS Data. *PLoS ONE* 7, e45474

2. Khatri, K., Staples, G. O., Leymarie, N., Leon, D. R., Turiák, L., Huang, Y., Yip, S., Hu, H., Heckendorf, C. F., and Zaia, J. (2014) Confident Assignment of Site-Specific Glycosylation in Complex Glycoproteins in a Single Step. *J. Proteome Res.* 13, 4347–4355

3. Käll, L., Storey, J. D., MacCoss, M. J., and Noble, W. S. (2008) Assigning Significance to Peptides Identified by Tandem Mass Spectrometry Using Decoy Databases. *J. Proteome Res.* 7, 29–34

4. Bhatia, V. N., Perlman, D. H., Costello, C. E., and McComb, M. E. (2009) Software Tool for Researching Annotations of Proteins: Open-Source Protein Annotation Software with Data Visualization. *Anal. Chem.* 81, 9819–9823
